# Novel therapies for graft versus host disease with a focus on cell therapies

**DOI:** 10.3389/fimmu.2023.1241068

**Published:** 2023-10-05

**Authors:** Robert Zeiser, Olle Ringden, Behnam Sadeghi, Gil Gonen-Yaacovi, Oscar G. Segurado

**Affiliations:** ^1^ Department of Medicine at the University of Freiburg, Freiburg, Germany; ^2^ Department of Clinical Sciences, Karolinska Institute, Stockholm, Sweden; ^3^ ASC Therapeutics, Milpitas, CA, United States

**Keywords:** graft versus host disease, GvHD, Decidua stromal cells, DSC, allogeneic hematopoietic cell transplantation, cell therapy

## Abstract

Graft versus host disease (GVHD) can occur at any period post allogeneic hematopoietic stem cell transplantation as a common clinical complication contributing to significant morbidity and mortality. Acute GVHD develops in approximately 30-50% of patients receiving transplants from matched related donors. High doses of steroids are used as first-line treatment, but are unsuccessful in around 40% of patients, resulting in the diagnosis of steroid-refractory acute GVHD. Consensus has yet to develop for the management of steroid-refractory acute GVHD, and prognosis at six months has been estimated at around 50%. Thus, it is critical to find effective treatments that increase survival of steroid-refractory acute GVHD. This article describes the currently known characteristics, pathophysiology, and treatments for GVHD, with a special focus on recent advances in cell therapies. In particular, a novel cell therapy using decidua stromal cells (DSCs) was recently shown to have promising results for acute GVHD, with improved effectiveness over previous treatments including mesenchymal stromal cells. At the Karolinska Institute, severe acute GVHD patients treated with placenta-derived DSCs supplemented with either 5% albumin or 10% AB plasma displayed a one-year survival rate of 76% and 47% respectively. Furthermore, patients with steroid-refractory acute GVHD, displayed survival rates of 73% with albumin and 31% with AB plasma-supplemented DSCs, compared to the 20% survival rate in the mesenchymal stromal cell control group. Adverse events and deaths were found to be attributed only to complications of hematopoietic stem cell transplant and GVHD, not to the study intervention. ASC Therapeutics, Inc, in collaboration with the Karolinska Institute, will soon initiate a phase 2 multicenter, open-label study to further assess the efficacy and safety of intravenous DSC treatment in sixty patients with Grade II-IV steroid-refractory acute GVHD. This novel cell therapy represents a promising treatment to combat the poor prognosis that steroid-refractory acute GVHD patients currently face.

## 1 Introduction

Allogeneic hematopoietic cell transplantation (allo-HCT), using blood progenitor cells from a donor source, is a vital component in treating hematologic malignancies and other life-threatening cancers or nonmalignant disorders (1). Numbers of transplantations continue to grow worldwide, with almost 20,000 allo-HCT transplants reported by the European Society for Blood and Marrow Transplantation (EBMT) in 2019 (2) and over 9,000 transplants in the United States in the same period according to the Center for International Blood and Marrow Transplant Research (CIBMTR) (3–5). The cost burden for these patients, especially if they develop complications, can be quite high. Recently outpatient allografting has become possible, lowering the cost, particularly due to a reduction in overnight hospital stays. However, complications and their subsequent treatments are still common and lead to significantly higher financial burdens.

Graft-versus-host disease (GVHD) is the most common life-threatening complication after allo-HCT (6, 7). Among adult matched related donor transplant recipients (US, 2017-2018), 13% of deaths at or after 100 days of transplantation were attributed to GVHD (3).The two main clinical presentations of GVHD are acute (aGVHD) and chronic (cGVHD). Consensus criteria define aGVHD and cGVHD based on clinical characteristics and time of onset relative to the transplantation. According to experts from the EBMT, CIBMTR, and National Institutes of Health (NIH), the most comprehensive and detailed criteria available for the diagnosis and scoring of GVHD are the Mount Sinai Acute GVHD International Consortium (MAGIC) criteria for aGVHD (8) and the NIH 2014 criteria for cGVHD (9, 10).

## 2 Graft versus host disease characteristics and manifestations

Acute GVHD develops in 30-50% of patients after allo-HCT from a matched related donor (6, 11–14). Classically, the onset of aGVHD appears within 100 days of transplant, although late-onset aGVHD can occur after 100 days. The disorder typically presents as an acute inflammatory syndrome primarily affecting the skin, gastrointestinal tract, and liver. Affected patients have a maculopapular rash that starts around the neck and shoulders and often involves the palms, soles, and ears (6). Gastrointestinal (GI) manifestations include abdominal cramping and pain, diarrhea, hematochezia, and ileus (lower GI), as well as anorexia, nausea, and vomiting (upper GI). Severity is determined by the volume of diarrhea, which is secretory and may persist despite cessation of oral intake. In addition, there is increasing evidence that organs with less apparent, acute damage (or where drug toxicity may be a differential diagnosis) may be targets of aGVHD, including the central nervous system, lungs (15), ovaries and testis, thymus, bone marrow, and kidney (16). aGVHD is clinically graded and staged in severity from Grades I to IV, depending on the involvement and extent of the skin, liver, upper gastrointestinal tract, and gut. The overall clinical grade is based on the organ with the most severe damage (8).

cGVHD is the leading cause of late morbidity in recipients of allo-HCT and is associated with a higher risk of non-relapse mortality (17, 18). The disorder is diagnosed in 30% to 70% of patients after allo-HCT and most often develops between 100 days and one-year post-transplantation, but 5% to 10% of affected patients do not develop signs and symptoms until later. Approximately 30% of cGVHD is de novo without any preceding aGVHD (19–22). Symptoms and manifestations of cGVHD are heterogeneous and pleomorphic; it can affect any organ and is characterized by a gradual onset involving tissue inflammation and fibrosis that often results in permanent organ dysfunction (22–24).

### 2.1 Pathophysiology

GVHD occurs when donor T cells recognize the host tissues as foreign due to histocompatibility differences between the donor and host. Even with matching major histocompatibility complex antigens, many patients develop GVHD because of minor histocompatibility antigen differences lying outside of the human leukocyte antigen (HLA) loci (14, 25, 26). Donor T cells from the allografts are critical for successful transplantation as they are essential for hematopoietic engraftment, reconstitution of T cells immunity, and development of potent beneficial antitumor effect (graft-versus-tumor effect), which precludes complete abrogation of donor immune responses (27–29).

The pathophysiology of aGVHD includes an initial stage of tissue damage from the conditioning regimen leading to activation of host antigen-presenting cells (APC) by danger-associated molecular patterns (DAMPs), such as ATP (30) and pathogen-associated molecular patterns (31). In addition, loss of microbial diversity and metabolites thereof leads to loss of epithelial and immune homeostasis. In the second stage, donor T cells are activated in response to alloantigen expressed on host or donor APCs. T cells proliferate and differentiate into T helper (Th) 1 and Th17 cells, which are involved in the activation of cluster of differentiation (CD) 4 cytotoxic T lymphocyte (CTL), CD8 CTL, and natural killer (NK) cells that mediate tissue damage. In the third stage, effector T cells, together with proinflammatory cytokines, attack the epithelial cells of the skin, liver, lung, and gastrointestinal tract (6, 32–34).

As with aGVHD, damage to host tissue and inflammatory cytokine release occurs due to pretransplant conditioning in the first phase of cGVHD. The second stage in the pathogenesis of cGVHD is suggested to include thymic injury and T and B cell dysregulation, followed by a third stage of aberrant tissue repair, often with fibrosis (23, 24, 35, 36). While aGVHD demonstrates an exacerbated inflammatory mechanism that is mainly caused by the presence of proinflammatory cytokines and activated donor T cells, cGVHD displays autoimmune features involving alloreactive and dysregulated T and B cell interactions with macrophages, dendritic cells (DCs), and neutrophils, ultimately initiating profibrotic pathways (35, 37). Recently, two drugs have been approved by the US Food and Drug Administration (FDA) for the treatment of cGVHD, namely, ibrutinib and belumosudil (38), and one drug, ruxolitinib, was approved by both the FDA (https://www.fda.gov/drugs/resources-information-approved-drugs/fda-approves-ruxolitinib-acute-graft-versus-host-disease) and by the European Commission (https://www.ema.europa.eu/en/medicines/human/EPAR/jakavi) for the treatment of Steroid refractory (SR) aGVHD.

### 2.2 Current treatment options

The standard backbone of GVHD prevention in patients undergoing allo-HCT includes the combination of a calcineurin inhibitor (cyclosporine or tacrolimus), which reduces the expansion of effector T cells by blocking interleukin (IL)-2, with a short course of methotrexate, which interferes with alloreactive T cell division (39–41). In December 2021, the FDA approved the selective T cell co-stimulation modulator, Abatacept, for the prophylaxis of aGVHD, combined with a calcineurin inhibitor and methotrexate in adult and pediatric patients (42).

In cases of aGVHD occurrence, the recommended first-line treatment is systemic high-dose steroid therapy. Of note, it is important to control GVHD as profound immunosuppression can lead to relapse of the original disease and increase the risk for opportunistic infections (11). Currently, first-line treatment, according to the EBMT guidelines, consists of corticosteroids at 1-2 mg/kg per day for aGVHD Grade 2-4 (43–46). However, less than 50% of patients achieve durable responses (39, 44). In addition, approximately 40% of patients do not respond to corticosteroid therapy and are diagnosed with steroid-refractory (SR)-aGVHD (47). SR-aGVHD can be defined as disease progression following 3 to 5 days of treatment or no response following 5 to 7 days of treatment; however, the exact definition can vary by treatment center. The reported 6 month survival estimate for patients with SR-aGVHD is approximately 50%, with 30% or less surviving beyond two years (14, 44, 48, 49).

There is no consensus regarding the optimal approach for the management of SR-aGVHD; as such, there is no accepted standard of care treatment (47, 50, 51). The American Society for Blood and Marrow Transplantation (ASBMT), the joint working group established by the British Committee for Standards in Haematology, and the British Society for Bone Marrow Transplantation outlined the options for SR-aGVHD therapy, which include extracorporeal photopheresis (ECP), anti-tumor necrosis factor (TNF)-α antibodies, anti–IL-2 receptor antibodies, mechanistic target of rapamycin (mTOR) kinase inhibitors, mycophenolate mofetil, methotrexate, alemtuzumab, antithymocyte globulin, and etanercept (44, 47, 51, 52). Unfortunately, most treatment options for GVHD can be quite costly with newly developed cell therapies being no exception. In general, most companies do not report the cost of stem cell therapies but have estimated in 2021 to range between $1,200-$28,000 per treatment (53). Costs associated with SR-aGVHD are particularly high, primarily because of the long hospitalization time, but even the common treatments vary dramatically depending upon which treatment is chosen, whether multiple doses are necessary, and the location that the treatment is given.

As mentioned previously, GVHD prophylaxis commonly involves a calcineurin inhibitor combined with methotrexate. Meta-analysis of methotrexate for SR-aGVHD discovered an overall response rate of approximately 70% with a 59.2% complete response (54). Methotrexate is a relatively cheap option as far as treatments for GVHD; the six month cost in 2018 was reported to be around $200 (55). Another common, but much more costly treatment, ECP, has been approved by the FDA since 1988, originally for the treatment of Sezary syndrome, a leukemic cutaneous T-cell lymphoma (56). The use of ECP for GVHD has been studied since the 1990s. This procedure involves the separation of mononuclear cells from plasma, exposure of the cells to UVA irradiation and 8-methoxypsoralen, and reinfusion. The mechanism of action involves lymphocyte apoptosis and differentiation of dendritic cells, leading to the increase of anti-inflammatory cytokines, decrease of pro-inflammatory cytokines, and promotion of regulatory T-cell generation (56). ECP treatment costs range from around $500 to over $100,000, with the median price being reported in 2021 as approximately $9,000 (57).

Alemtuzumab and human chorionic gonadotropin are cost-effective treatment options particularly in low and middle income countries. In fact, the Campath Distribution Program provides it free for patients if found to be the necessary treatment (58). Alemtuzumab is a monoclonal antibody treatment that reduces the number of immunocompetent T-cells and is shown to reduce the incidence of GVHD and mortality post-transplant. This anti-CD52 monoclonal antibody treatment has shown response rates above 60% in several clinical trials for aGVHD (59–62). Human chorionic gonadotropin as an adjunct therapy for SR-aGVHD, which may be effective due to its tissue regeneration abilities by enhancing the circulating levels of epidermal growth factor. This treatment also has shown a partial or complete response rate above 60% in clinical trials, although the optimal dose is still under investigation (63, 64). Because this drug is commercially available and inexpensive, averaging $296 per vial in 2019 (65), it is a promising option for middle and low income patients.

As aGVHD patients typically present with increased TNF-α levels, another effective anti-inflammatory treatment is the use of anti-TNF-α antibodies. However, the lack of efficacy in a portion of patients and the potential for life-threatening infections have reduced its use in treating SR-aGVHD (66). Infliximab and etanercept both target TNF-α. However, etanercept seems to be effective in skin and gut GVHD, but not hepatic GVHD (67). Annual treatment for TNF blockers varies, with the 2013 annual costs per patient ranging from $14,000-$25,000. Etanercept had the lowest cost, followed by adalimumab and infliximab (58). Although mycophenolate mofetil is effective and well-tolerated, it also causes high-risk of infection (68). Toxicity was also a concern for this treatment, and a previous trial identified it to be effective for chronic GVHD but suboptimal for aGVHD. This treatment works by inhibiting T and B lymphocyte proliferation and costs in 2016 reported to be approximately $1,000 per month (69). mTOR signaling is enhanced in GVHD, making mTOR a promising target for GVHD treatment (70). The first clinical trials for mTOR inhibitors as second-line therapy resulted in significant side effects and toxicity, requiring better optimization of dosing and further safety and efficacy trials (70). However, in 2020 sirolimus, an mTOR inhibiting drug, was compared to low-dose prednisolone treatment, revealing that sirolimus had superior complete and partial response rates (71). 2016 costs for mTOR inhibitors ranged from $1000-$2000 per month (69), with the 2018 six month cost being estimated at $6,000 (55).

The use of anti-IL-2 receptor antibodies have varied in effectiveness between the four currently commercially available drugs (72). The IL-2 receptor is targeted because of its expression on T-lymphocytes. The varying efficacy may be due to the different half-lives that range from approximately 70 minutes to 25 days or other factors in their production. According to a meta-analysis, the response rate was highest for basiliximab (72). Basiliximab was estimated to cost around $3,000 per dose in 2016 (69). Although IL-2R antibodies, antithymocyte globulin, infliximab, and etanercept have yet to show increased survival compared to single agent steroids, they are useful in steroid-refractory patients (54). Antithymocyte globulin targets antigen-expressing cells such as T-cells, B-cells, macrophages, NK cells, and dendritic cells, which is used in the prevention of severe aGVHD (73). This treatment was estimated to cost around $3,000 per dose as of 2016 (69). This 2016 article reports that alemtuzumab is the least costly induction agent followed by basiliximab (anti-IL-2 receptor antibody) and antithymocyte globulin. Treatment costs may have substantially changed since these average US reports in 2013 and 2016; however, the relative differences between treatments are likely similar.

In addition, ruxolitinib, a selective Janus kinase 1/2 inhibitor, was approved by the FDA and the European Commission for the treatment of SR-aGVHD in adult and pediatric patients (≥12 years old). Based on results from the REACH-1 study, the median duration of response from ruxolitinib treatment was 0.5 months. The median time from the day‐28 response to either death or the need for a new therapy for aGVHD was 5.7 months (74–76). In a randomized, Phase 3 trial for SR-aGVHD testing nine treatments, ruxolitinib demonstrated a higher overall response compared to other treatments. However, ruxolitinib was unable to produce significant changes in survival or non-relapse mortality (77). Thus, there is still an unmet need in terms of greater efficacy and increased long-term survival for SR-aGVHD patients. The cost of this treatment for six months in 2018 was approximately $83,000 (55).

Treatment of cGVHD typically requires the prolonged (median 2 to 3.5 years) use of immunosuppressive agents. First-line treatment includes high-dose corticosteroids, typically 0.5 to 1 mg/kg per day. An approximate 50% response rate is seen with steroids; second-line therapy is required in more than half of patients within two years (78). The heterogeneous manifestations of cGVHD make clear guidelines for second-line treatments necessary (24, 43, 79)..

Ibrutinib, a tyrosine kinase inhibitor targeting Bruton’s tyrosine kinase, was the first FDA-approved treatment for cGVHD (approved in August 2017), demonstrating an overall response rate (ORR) of 67% in patients with SR-cGVHD (80, 81).The cost of six months of treatment in 2018 was approximately $80,000 (55). In July 2021, the FDA approved belumosudil, a kinase inhibitor, for adult and pediatric patients twelve years and older with cGVHD after the failure of at least two prior lines of systemic therapy. The best response rate for belumosudil at 200 mg twice daily was 77%, and symptom reduction was reported in 62% of patients (82). Belumosudil taken once daily was reported in a 2022 article to cost around $232,000 per year (83). In September 2021, approval of ruxolitinib was expanded to include treatment for cGVHD in adult and pediatric patients ages twelve and older after failure of ≤2 lines of systemic therapy with almost 50% ORR and median failure-free survival of >18 months demonstrated in the REACH3 Phase 3 study (16, 84). The 2020 NIH cGVHD Consensus Development Project was established in order to address gaps and needs in cGVHD research in the coming years (20, 85, 86).

Recent research has placed significant importance on the microbiota in various diseases, including for GVHD. At least six studies and five case reports of fecal transplantation have been published as of 2022 (87). Overall, this seems to be a safe and effective treatment strategy for GVHD. Pooling of these studies found complete remission in around 56% of patients with limited treatment-related mortality or adverse effects. Studies have focused on GI-related aGVHD, many being steroid-refractory. When fecal microbiota transplants were investigated for intestinal GVHD in 2020, a complete response and restoration of microbial diversity in the gut resulted within a month in ten of the fifteen patients (88). As this treatment is still in clinical trials for GVHD, the cost is not yet determined.

## 3 Cell therapy for GVHD

Several cell types can be utilized to suppress alloreactive immune cells. Mesenchymal stromal cells (MSCs) and, to a lesser degree, regulatory T cells (Treg) have been efficacious in human immune reactions (86). Tregs are CD4+ T cells that express the transcription factor, forkhead box P3 (FoxP3), resulting in anti-inflammatory pathway initiation. Early-phase clinical trials using Treg therapy have supported its feasibility and tolerability, but more research is necessary to demonstrate the efficacy and reproducibility of this approach in late-stage randomized clinical studies. Current obstacles in Treg cell therapy for GVHD include the high dose requirement of effective polyclonal Tregs for preventing or treating GVHD and the challenges surrounding ex vivo Treg expansion (89, 90), as well as production cost. This review will focus on stromal cell therapies for GVHD.

### 3.1 Mesenchymal stromal cells

The non-hematopoietic human MSC has the unique ability to differentiate into various mesodermal (osteocytes, adipocytes, and chondrocytes), ectodermal (neurocytes), and endodermal (hepatocytes) lineages (91). MSCs can be obtained from various adult or neonatal tissues such as peripheral blood, bone marrow (BM), and adipose tissue or umbilical cord, amniotic membrane, and placenta. Placenta derived tissues pose a significant advantage over adult tissues as invasive procedures and certain ethical considerations are not necessary (92–95).

MSCs have emerged as the most studied cell type for clinical and experimental cell therapy for various indications, including GVHD (96–98). The cells contain high plasticity, self-renewal, immunomodulatory, and anti-inflammatory properties. In addition, MSCs minimally produce host immune responses since they do not express HLA class-II and costimulatory molecules. Allogeneic MSCs could be used as an effective treatment for acute inflammatory disorders since their effects utilize hit-and-run mechanisms. In contrast to pharmacological immunosuppressive drugs, MSCs have very little, if any, side effects or toxicity, and long-term adverse effects are not expected (99, 100).

Multiple factors and mechanisms are involved in MSC-mediated immune modulation and regenerative function. They include paracrine activity, which involves the secretion of proteins/peptides and hormones, as well as mitochondrial transport via tunneling nanotubes or microvesicles and transport of exosomes or microvesicles (101). Exosomes and microvesicles are vehicles for the transport of DNA, RNA, and other molecules. MSCs promote an immunosuppressive environment by releasing immunomodulatory factors and cytokines, such as indoleamine 2,3-dioxygenase (IDO), prostaglandin E2 (PGE2), IL-10, transforming growth factor-β, nitric oxide, HLA-G5, and TNF-α-induced gene/protein 6, which assist in directing the phenotype, function, and homing of immune cells. MSCs may suppress T cell proliferation, proinflammatory cytokine release, cytotoxicity, and Th1/Th2 balance and also affect B cell viability and antibody secretion (102–106). All possible mechanisms of the immunosuppressive activity of MSCs in GVHD have been previously reviewed in detail (107–109).

The first case report of MSCs used for the treatment of GVHD was reported from the Karolinska Institute in Sweden (110), followed by promising results published by the European Group for Blood and Marrow Transplantation Developmental Committee in 2008. Patients with a complete response to MSC treatment had lower transplantation-related mortality one year after infusion (11 [37%] of 30) than patients with partial or no response (18 [72%] of 25; p = 0.002) in 55 patients with severe SR-GVHD (111). Subsequently, numerous clinical studies have been carried out worldwide to investigate the safety and efficacy of MSCs in immunomodulatory cell therapies during HCT to prevent and treat GVHD, repair damaged tissue, and facilitate hematopoietic stem cell engraftment (96, 97, 99, 105, 108, 112, 113). The source of MSCs in these studies was mainly BM and umbilical cord blood (UCB). Various doses (between 10^5 and 10^7 cells/kg) were administered in a single dose or multiple doses in heterogeneous cohorts of pediatric and adult patients. MSC treatment was well-tolerated and safe for patients; however, there is no definitive evidence of efficacy, and responses were unpredictable. Published systematic reviews and meta-analyses could not conclude that administration of MSCs from BM or UCB is effective in the prevention or treatment of aGVHD, and no significant effect was observed on relapse rate or overall survival in patients after allo-HCT (99, 112, 114–116). The lack of patients’ stratification criteria, specific biomarkers, and heterogeneity of MSC preparations may have contributed to the lack of conclusive evidence in clinical studies with MSCs. Data collected by the EBMT centers highlight the variability in MSC manufacturing as clinical products and the need for coordination (117).

There are currently no FDA-approved MSC therapies on the market in the US. However, several MSC products have been granted regulatory approval in other countries (Korea, Canada, Japan, New Zealand) for the treatment of various conditions, including aGVHD (118, 119).

Remestemcel-L (Ryoncil™, Mesoblast, Ltd; formerly Prochymal^®^, Osiris Therapeutics Inc.), a human BM-derived MSC product, was well tolerated in clinical studies with no identified infusion-related toxicities or other safety concerns (120–125). It was approved for use in Canada and New Zealand in 2012 for treating SR-aGVHD in pediatric patients based on the safety profile of the product and promising evidence of efficacy in the subgroup of pediatric patients (126).

Remestemcel-L was not approved for use by the FDA as it failed to show superiority over the placebo in a randomized, Phase 3 clinical study in patients with SR-aGVHD (121). The study did not meet the primary endpoint of greater durable complete response (DCR, defined as completely resolved aGVHD symptoms for at least 28 days after beginning treatment) in the remestemcel-L group compared with placebo (35% versus 30%; p = 0.42). Post hoc analyses found that patients with liver involvement who were given one or more infusion(s) of remestemcel-L had a higher DCR and higher overall complete or partial response rate than those given a placebo (29% versus 5%; p= 0.047). Among high-risk patients (aGVHD grades III-IV), remestemcel-L demonstrated a significantly higher overall 28-day response compared to placebo (58% versus 37%; p= 0.03). Furthermore, pediatric patients had a higher overall response with MSCs compared to placebo (64% versus 23%; p = 0.05).

Temcell^®^ (JCR Pharmaceuticals Co. Ltd), a manufactured MSC product equivalent to remestemcel-L, was approved in Japan to treat patients of all ages with acute GVHD. Within the first three years after approval, Temcell was evaluated in 381 patients. The ORR was 61% for the 151 evaluable patients who received it as second-line therapy following first-line steroid therapy for aGVHD (127).

As summarized in **Table 1**, there are currently eleven clinical studies using MSCs for the treatment or prevention of GVHD registered at ClinicalTrials.gov (with status of recruiting, not yet recruiting, or active not recruiting), with the majority being early-stage studies. One Phase 3 study of MC0518 (Medac GmBH, Germany) is currently recruiting adult and adolescent subjects with SR-aGVHD in 34 centers across Europe. MC0518 (other names: MSC-FFM, Obnitix) is an allogeneic MSC treatment product for SR-aGVHD, created by pooling the BM mononuclear cells of eight unrelated, healthy donors (128, 129). The manufacturing protocol of MC0518 is characterized by high potency and near-identical individual doses. Compared to other MSC products in clinical trials, MC0518 is relatively young, harvested at passage 3. Trials reveal good clinical tolerability of MC0518 treatment in 69 patients (51 children and 18 adults) with refractory aGVHD Grade II-IV, showing 83% ORR at day 28 and a 71% survival probability rate at six months (130).

**Table 1:** Overview of current clinical studies using MSCs for the treatment of GVHD.

Sponsor, Country	Product	Phase Status	Planned Participants	Study Title (ClinicalTrials.gov Clinical Trial Number) EudraCT Number (if available)
Medac GmbH, **Germany**	MC0518	Phase 3 Recruiting	210	A Randomised, Open-label, Multicentre, Phase 3 Trial of First-line Treatment with Mesenchymal Stromal Cells MC0518 Versus Best Available Therapy in Adult and Adolescent Subjects with Steroid-refractory Acute Graft-versus-host Disease After Allogeneic Haematopoietic Stem Cell Transplantation (IDUNN Trial) (NCT04629833) EudraCT Number: 2019-001462-15
University of Liege, **Belgium**	MSCs	Phase 2 Recruiting	100	Infusion of Mesenchymal Stem Cells as Treatment for Steroid-Resistant Grade II to IV Acute GVHD or Poor Graft Function: a Multicenter Phase II Study (NCT00603330) EudraCT Number: 2007-004310-14
Andalusian Initiative for Advanced Therapies, **Spain**	Adult Allogeneic MSCs from adipose tissue	Phase 1/2 Active, not recruiting	16	Clinical Trial Phase I/II Graft Versus Host Disease Treatment Refractory to First-line Therapy with Sequential Infusion of Mesenchymal Cells Allogeneic Expanded Adipose Tissue in Vitro (NCT02687646) EudraCT Number: 2014-005533-32
MD Anderson Cancer Center, **USA**	Cord blood tissue-derived MSCs	Pilot study Recruiting	24	A Randomized Controlled Pilot Study of Two Doses of Cord Blood Tissue-Derived Mesenchymal Stromal Cells Combined with Ruxolitinib Versus Ruxolitinib Alone for Therapy of Steroid-Refractory Acute Graft Versus Host Disease (NCT04744116)
Edwin Horwitz, Emory University, **USA**	IFNγ-primed human BM-derived MSCs	Phase 1 Recruiting	45	Interferon γ-Primed Mesenchymal Stromal Cells as Prophylaxis for Acute Graft v Host Disease After Allogeneic Hematopoietic Cell Transplantation for Patients with Hematologic Malignancies and Myelodysplasia (NCT04328714)
University of Kansas Medical Center, **USA**	MSCTC-0010 UC Wharton’s jelly MSCs	Phase 1 Recruiting	10	A Phase I Study to Evaluate the Safety of Umbilical Cord - Derived, Ex-Vivo Cultured and Expanded Wharton’s Jelly Mesenchymal Stem Cells for the Treatment of De Novo High Risk Acute or Steroid Refractory Acute Graft Versus Host Disease (NCT03158896)
Peking University People’s Hospital, **China**	Umbilical cord MSCs and anti-CD25 mAb	Phase 3 Not yet recruiting	130	Efficacy and Safety of UC-MSCs for the Treatment of Steroid-resistant aGVHD Following Allo-HSCT: A Multicenter, Randomized, Open-label Trial (NCT04738981)
Nanfang Hospital of Southern Medical University, **China**	MSCs	Phase 2 Recruiting	152	Mesenchymal Stem Cell for Treatment of Chronic Graft-versus-host Disease After Allogeneic Hematopoietic Stem Cell Transplantation (NCT04692376)
Shenzhen University General Hospital, **China**	UC MSCs	Phase 1/2 Recruiting	10	Clinical Trial of Umbilical Cord Mesenchymal Stem Cells in the Treatment of Moderate/Severe Chronic Graft-versus-host Disease (NCT05152160)
Cytopeutics Sdn. Bhd., **Malaysia**	Cytopeutics^®^ UC-derived MSCs	Phase 1/2 Recruiting	40	Cytopeutics^®^ Umbilical Cord Mesenchymal Stem Cells (Cyto-MSC) for Patients with Grade II-IV Acute Graft-Versus-Host Disease: A Phase I/II Clinical Study (NCT03847844)
SCM Lifescience Co., LTD., **Korea**	SCM-CGH (BM MSCs)	Phase 2 Recruiting	77	A Multicenter, Randomized, Parallel Group, Double-blind, Phase 2 Trial to Evaluate Efficacy and Safety of SCM-CGH in Patients with Steroid-Refractory or Dependent Chronic Graft-Versus-Host Disease (NCT04189432)

Source: www.clinicaltrials.gov (date accessed: 14 February 2022).

#### 3.1.1 Biomarkers of MSCs in GVHD

Biomarkers are required as an assessment tools to predict clinical response and monitor the efficacy of therapy. GVHD patients’ serum has been used to identify biomarkers related to tissue damage during the pathogenesis of GVHD (131–133) Acute GVHD biomarkers include plasma levels of IL-2 receptor subunit α (IL-2Rα), TNF receptor 1 (TNFR1), IL-8, hepatocyte growth factor (134, 135), and amphiregulin (136). Organ-specific biomarkers include cytokeratin fragment 18 (CK18) for intestinal and hepatic GVHD (137), suppression of tumorigenicity 2 (ST2) and regenerating islet-derived 3α (Reg3α) for gastrointestinal GVHD (138–140), and elafin for skin GVHD (141). The gastrointestinal biomarkers ST2 and Reg3α are also included in the MAGIC algorithm probability (MAP) that estimates the probability of 6-month non-relapse mortality for individual patients (142, 143).

Despite the importance of the above-mentioned plasma biomarkers in GVHD, their role in predicting or monitoring MSC response has not been confirmed (116). Decreases in plasma biomarkers such as Reg3α, CK18, TNFRI, IL-2Rα, and elafin were observed in patients who responded to MSCs (144–146). However, another study found that Reg3α and IL-2Rα were not correlated with the response to MSCs in aGVHD patients (144). ST2 has been reported to be a strong predictive marker for patients unresponsive to GVHD therapy (139). However, in a Phase 2 study of 48 patients with SR-aGVHD, ST2 was not predictive of therapy resistance before infusion of MSCs (147). In addition, neither the absolute numbers nor the frequencies of CD8 and NK cells seem to have a role in predicting MSC response (147).

In a study that assessed three proposed aGVHD serum markers (Reg3α, CK18F, and elafin) and the lymphocyte profiles of 16 aGVHD patients given MSC treatment, no obvious markers for MSC therapy response were revealed (148). In a cohort of 40 pediatric SR-aGVHD patients treated with remestemcel-L, the baseline biomarker profile consistently displayed inflammation including increases in the activation of CD4+ and CD8+ T cells and plasma levels of Reg3α and ST2. Over the course of the study, MAP was significantly decreased from baseline through 180 days, which was attributable to significant reductions in ST2 levels. In addition, levels of activated T cells declined, and levels of T, B, and NK cells generally increased over time (149).

In a small cohort of SR-aGVHD patients, the increase of serum PGE2 after MSC treatment was significantly greater in the responders compared to the non-responders. Hence, it was suggested that PGE2 monitoring could estimate the immunological activity of MSC therapy in GVHD patients (150). In addition, it was recently reported that MSCs, infused in the presence of cytotoxic cells, experience caspase activation and apoptosis, which is shown to be required for their immunosuppressive function. Cytotoxic assays have been found to predict the clinical responses in murine models and patients with GVHD; patients who demonstrate high cytotoxicity display relatively good responses to MSC therapy, while improvements were not found in patients given MSC infusion who displayed low or absent cytotoxic activity (151, 152). While the recipient’s cytotoxic cell activity is required for MSC therapeutic efficacy and could help identify ideal patients who will likely respond well to MSC therapy, PGE2 levels could be a biomarker for monitoring responses and detecting early treatment failures after MSC infusion (116).

### 3.2 Placenta-derived decidua stromal cells

Placenta-derived decidua stromal cells (DSCs) are a type of stromal cells of maternal origin isolated from the fetal membranes of term placenta. The placenta includes the amnion, chorionic plate, villous and smooth chorion, decidua basalis, and umbilical cord. The decidua is a maternal uterine tissue that plays an important role in maternal-fetal immune tolerance (153, 154). DSCs have better expansion capacity and stronger immunomodulatory effects compared to stromal cells originating from fetal tissues, amnion, and chorion and BM-MSC (155).

Non-clinical studies have shown that placenta-derived DSCs differ from BM-MSCs in several aspects. They are smaller in size, show less differentiation capacity for chondrogenic and osteogenic fates, have a stronger inhibitory effect on allogeneic T cell proliferation, and in vitro data shows that they promote coagulation more effectively than BM-MSCs (156–162). Furthermore, studies of human DSCs showed that, unlike MSCs, DSCs have a similar inhibitory capacity on the proliferation of T cells whether fresh or frozen and thawed, and cell viability was maintained for 24 hours in both circumstances (163).

In vitro evaluation of DSCs shows a great immunosuppressive capacity for the proliferation of alloreactive T cells, production of proinflammatory cytokines, and induction of anti-inflammatory IL-10 secretion (158). In addition, these cells suppress the production of interferon-gamma (IFN-γ) and IL-17, express high levels of integrins that may be important in targeting inflamed tissues, and express high levels of adhesion markers compared to other types of stromal cells (158).

Erkers et al. (2013) reports that DSCs need to be near alloreactive lymphocytes to mediate a suppressive effect via paracrine mechanisms or increase the frequency and/or expression level of the Treg population. Thus, DSCs may not use paracrine factors solely for systemic immunosuppression but more specifically to target T cells directly in affected tissues (157). DSCs inhibit dendritic cell differentiation and their ability to induce allogeneic T cell proliferation; IDO and PGE2 mediate the inhibitory effect (161). Blocking the activity of IDO, PGE2, programmed cell death ligand 1, and IFN-γ impaired the antiproliferative ability of the DSCs in mixed lymphocyte reactions (157).

DSCs may influence stromal cell-mediated immune modulation and changes in activated T cell phenotypes through IL-2 production and IL-2R signaling. Sirolimus and cyclosporine A target the IL-2 signaling pathway, diminishing the antiproliferative effect of DSCs in vitro (164). The characteristics of DSCs are summarized in **Table 2**.

**Table 2:** Characteristics of DSCs.

Positive for	CD29, CD73, CD90, CD105, CD44, CD49d, HLA class-I antigens
Negative for	CD11, CD19, CD34, CD31, CD45, epithelial cell markers, and HLA class-II antigens
Increase expression of	IL-10, integrins, PDL-1/-2, Tregs
Decrease expression of	IFN-γ, IL-17
Suppress	Immune response

**Figure 1:**
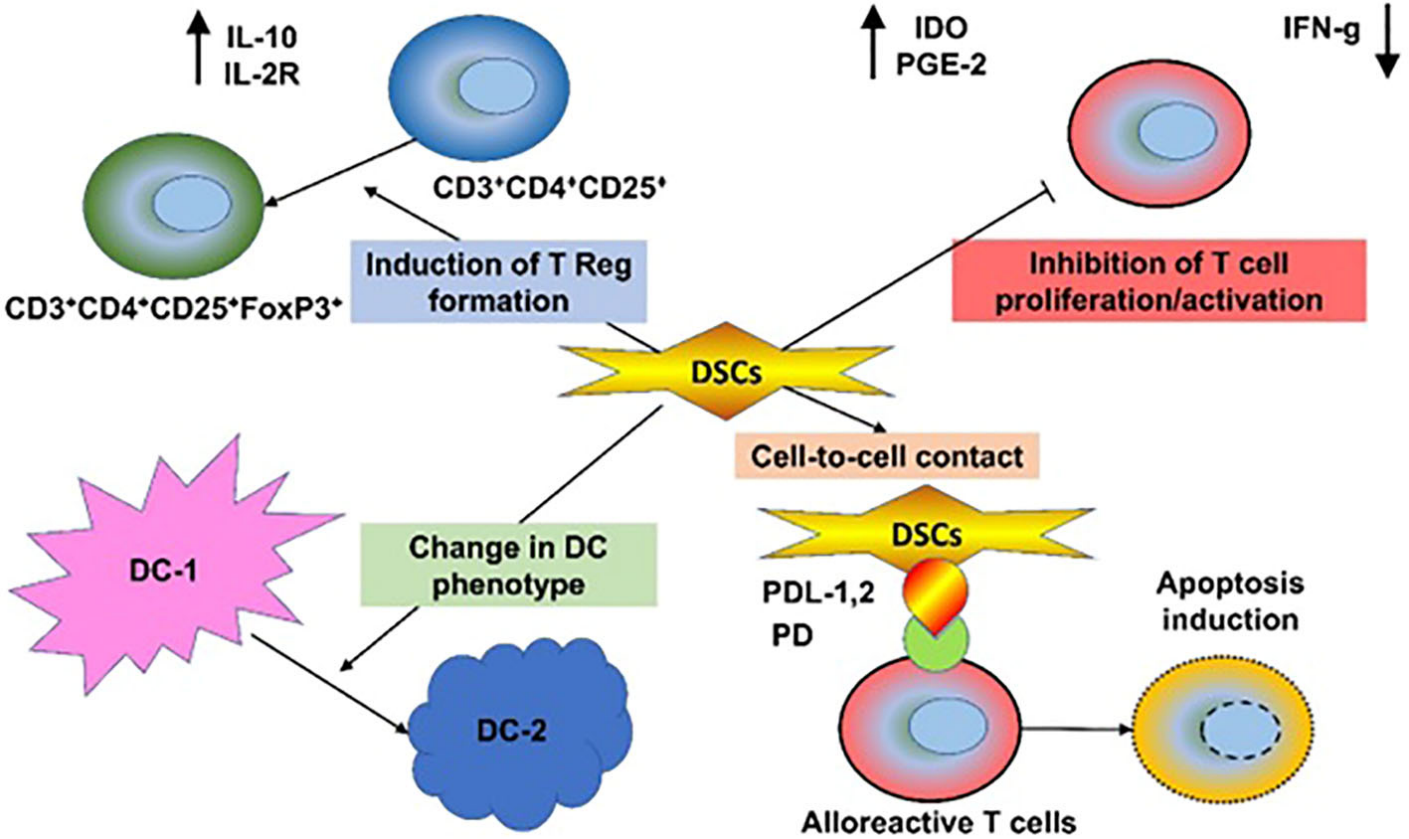
Immunomodulatory and Regenerative Properties of DSCs. Decidua stromal cells (DSCs) employ a broad array of immunomodulatory mechanisms, produced directly through intrinsic or secreted immunomodulatory and regenerative molecules, such as Galectin-1, indoleamine-2,3-dioxygenase (IDO), or indirectly via changes in innate and adaptive immune cells, involving inhibition of T and NK cells and induction of regulatory T cells, polarization of T helper cells, dendritic cells, monocytes, and macrophages to type 2 phenotype, and inhibition of B cell maturation. This results in benefits to the cytokine profile, including downregulation of the proinflammatory molecules TNF-α, IFN-γ, perforin, and granzyme and secretion of anti-inflammatory IL-10 and multiple trophic and regenerative factors. This process results in beneficial tissue repair.

Comprehensive non-clinical short- and long-term toxicity studies of systemic DSC infusions were conducted in two animal models (rat and mouse) and in series of in vitro assays employing human blood (162). No thrombosis, organ damage, or toxicity were observed with doses up to 40 x 10^6 cells/kg (40 times higher than is used clinically). In vivo tracking of IV-infused DSCs has shown cell signals in the lungs of treated animals for up to four days post-infusion. Compared to BM-derived MSCs, the DSCs were smaller in size but showed stronger clotting ability in human blood and plasma in an in vitro assay. A heparin supplement was used to decrease in vitro clotting parameters and markers of MSC- and DSC-induced complement activation and coagulation, including thrombin-antithrombin complex and C3a (162). **Figure 1** displays the immunomodulatory and regenerative properties of DSCs.

A major obstacle in stromal cell therapy is the trapping of IV infused MSCs into the lung microvasculature, impairing their homing to target tissue due to the first-passage effect (the passive entrapment of MSCs in small vessels), which is a function of the cell size and deformability. DSCs have smaller diameters, being approximately half the volume of MSCs, which could encourage microvascular passage and reduce microvascular organ and pulmonary embolism risks (156, 165). In vitro data showed that DSCs have stronger hemostatic properties than MSCs, which could trigger stronger activation of the clotting system. However, none of our preclinical studies (162) or clinical application of DSCs (166, 167) supported this observation in vivo (156). Thus, to address any possible risk, we optimized the cell graft preparation and infusion process using heparin (166, 167).

#### 3.2.1 Academic studies with placenta-derived DSCs

DSCs have been clinically investigated for the treatment of severe aGVHD at the Karolinska Institute in Sweden (155, 166–169), as well as cGVHD (170), hemorrhagic cystitis (171), acute respiratory distress syndrome (ARDS) (171), lung insufficiency following Covid-19 (172) and radiculomyelopathy (173), which demonstrated positive responses in the vast majority of patients. DSCs, like other cells, first go to the lungs after IV infusion due to pulmonary trapping (165, 174, 175). Due to this, DSCs are a promising therapy for lung-related disease such as ARDS and COVID-19. Three cases of ARDS, a rare side effect of HCT, have been treated with MSCs. Two patients given BM-MSCs did not survive, although the third patient who received DSCs, showed a dramatic response, and has survived seven years post-treatment thus far (176). DSC therapy was also recently shown to be safe and capable of improving oxygenation, decreasing inflammatory cytokine levels, and clearing pulmonary infiltrates in patients with COVID‐19 (172).

The distribution of DSCs after IV infusion has also been investigated in severe cGVHD. The pilot study by Erkers et al. (2015) used DSCs labeled with 111Indium (111In), and the in vivo distribution was tracked for 48 hours in two patients. The labeled DSCs were initially located in the lungs, followed by dissemination to the liver and spleen (170). MSCs have been superior for use in treating acute rather than chronic GVHD, but DSC therapy may be more effective and needs further controlled trials to confirm the superiority over MSCs (177).

##### 3.2.1.1 DSCs in aGVHD

In the investigator-initiated study (Karolinska Institute Sweden; 170), a total of 38 subjects with severe aGVHD Grade 2-4 were enrolled and treated with placenta-derived DSCs, as a second-line therapy. Of the subjects, 21 (ages 1.6-72.4 years) received DSCs supplemented with 5% albumin (median dose 1 x 10^6 cells; DSC/Albumin group at inclusion), and 17 (ages 0.9-65.6 years) received DSCs supplemented with 10% AB plasma (median dose 2 x 10^6 cells; DSC/AB Plasma group at inclusion). The number of patients who were steroid-refractory in the DSC/Albumin and DSC/AB Plasma groups was 11 and 13, respectively and were treated between 2011-2015. In both study groups, the majority of subjects were males (52.9% - 76.2%) and most subjects had aGVHD Grade 3 (71.4% in DSC/Albumin group, 88.2% in DSC/AB Plasma group). Control groups, including only patients with acute steroid-refractory GVHD, included 15 subjects receiving BM-MSC treatment (ages 34-65 years) and 32 historical controls receiving all available treatment as a second-line therapy except cells (ages 3.7-67.7 years). All control patients were from the Karolinska Institute Database, treated between the years 2000–2010, and were not matched to the treatment group.

The results reported in this review paper were taken from the manuscript Ringden et al. (167). The Fisher exact test was applied to test the statistical significance of the difference in the percentage of subjects achieving a treatment response between study groups in all aGVHD patients. Time to survival was determined with the Lifetable method using the log-rank (Mantel-Haenzel) test, taking censored data into account. The DSC/Albumin group had a higher chance of survival than the DSC/AB Plasma group with one-year survival rates of 76% and 47%, respectively (based on the Fisher exact test). A complete or partial response was found in all subjects (100.0%) in the DSC/Albumin group at four weeks after the first DSC dose (52.4% complete response, 47.6% partial response) compared with 58.8% in the DSC/AB Plasma group (29.4% complete response, 29.4% partial response) (p = 0.013). The cumulative incidence rate of GVHD-related mortality was significantly lower in the DSC/Albumin group compared to the DSC/AB Plasma group (death rates from aGVHD at one-year post-DSC treatment were 5% and 41%, respectively). No significant differences were observed between DSC groups in the incidence rate of chronic GVHD and hematological relapse.

In a subgroup of subjects with SR-aGVHD (n=71 in all groups; analysis is based on data from second-line therapy), the one-year survival rate aGVHD was significantly higher in the DSC/Albumin group (73%), versus the DSC/AB Plasma group (31%, p = 0.02), the MSC group (20%, p = 0.0015), and the historical control group (3%, p < 0.001), based on the Fisher exact test. A complete or partial response was observed in all SR-aGVHD subjects (100.0%) in the DSC/Albumin group at four weeks after the first DSC dose (63.6% complete response; 36.4% partial response), which was significantly higher than in the DSC/AB Plasma group and the MSC group with approximately only 25% seeing a response in the historical control group. The differences in response (complete and partial) vs. no response between the DSC/Albumin group and all other study groups were statistically significant at all timepoints evaluated.

The most common adverse events in the DSC groups were relapse (8/38 of subjects, 21%), fungal infection (6/38, 15.8%), and pneumonia (5/38, 13.2%). Lower rate of bacterial infection and multiple organ failure were reported in DSC groups versus control groups (7.9% and 34.4% respectively). Death events were reported for all study groups and occurred at lower rates in the DSC groups (44.7%) versus the control groups (96.9%). The majority of subjects in the DSC group died from either acute GVHD (23.7%), relapse (5.3%) or bacterial infection (5.3%); GVHD was the main cause of death in the control groups. Overall, based on the limited safety data available for analysis, no safety signals detected were attributable to the study intervention (DSC). The causes of adverse events and deaths were associated with common complications seen among subjects undergoing HCT and with severe aGVHD.

### 3.3 Industry-sponsored studies with placenta-derived DSCs (ASC930) and biomarkers of DSCs in GVHD

ASC Therapeutics, Inc. is developing ASC930, an allogeneic off-the-shelf investigational product consisting of live human placenta-derived DSCs in collaboration with the Karolinska team who originally developed DSCs as described. ASC930 is being developed for the treatment of patients with SR-aGVHD Grade II-IV following allo-HCT. ASC930 will be given to the hospitalized patients in an infused IV on a weekly basis. Each dose consists of 1 ± 0.2 x 10^6 cells/kg body weight and will be repeated for four weeks based on the patient’s condition.

The clinical development program of ASC930 was initiated with a re-analysis of safety and efficacy data from open-label studies conducted in academic settings (Karolinska Institute, Sweden) in subjects with aGVHD (166, 168, 178). An IND clearance has been received from the FDA, in addition to orphan drug designation, and a Phase 2 open-label multicenter study is planned to further assess the efficacy and safety of ASC930 treatment in 60 patients with SR-GVHD Grade II-IV (NCT04883918).

Due to the limited knowledge of how DSCs act in the body and how they activate the immune system, biomarkers are vital to determine the mechanisms of DSC treatments. Biomarkers for disease, immune, and cellular response to DSCs could help identify the optimal patients, treatment, and monitoring strategies for safety and efficacy of cell therapy for aGVHD. DSCs can be measured in the body, but also the donor-derived cell-free DNA can be quantified to understand the cellular actions or predict organ rejection. Inflammation also needs to be monitored because it can be a precursor to organ injury. Flow cytometry is the typical method for measuring the immune response by identifying and quantifying types of immune cells. Endothelial dysfunction biomarkers in the blood, such as ST2 and REG3α, can predict long-term outcomes and have been incorporated into the MAGIC algorithm probability to determine mortality post aGVHD treatment. High levels of these two biomarkers in the blood are found in aGVHD patients and predict mortality.

## 4 Conclusion

Steroid refractory acute GVHD is a life-threatening complication that limits the success of HCT. HCT is widely used in curing many hematological malignancies and other fatal disorders of the immuno-hematological system. The only FDA-approved drug for SR-aGVHD, ruxolitinib, cures just a portion of patients with SR-aGVHD. MSCs are rare cells in all body tissues but may be cultured and expanded to a large number of cells. Among several other features, MSCs are immunosuppressive and have been used to treat aGVHD, among other immunological disorders. MSCs are an attractive candidate for therapy because there are almost no reported side effects. DSCs have a greater immunosuppressive capacity than other sources of MSCs, and preliminary in vitro and clinical data suggest they more effectively treat alloreactivity and aGVHD compared to other sources of MSCs (bone marrow, fat, umbilical cord tissue, or amniotic fluid). Further clinical phase 1/2 trials and prospective randomized trials compared to the best available therapies, including ruxolitinib, are needed to establish DSCs as a therapy for SR-aGVHD.

## 5 Expert opinion

Finding safe and efficacious treatments for acute Graft versus Host Disease (aGVHD) after stem cell transplantation in patients with hematological malignancies is crucial because of its high mortality rate and the limited success of current treatment options. Over approximately two decades, autologous and allogeneic cell therapies have been used in clinical trials to treat aGVHD with encouraging responses. The introduction of decidua stromal cells (DSCs) has shown a 50% higher survival rate compared to those observed with mesenchymal stem cells (MSCs) in both pediatric and adult populations experiencing steroid-refractory aGVHD (SR-aGVHD). Interestingly, the vehicle used for DSCs was found to be relevant, since albumin (ASC930) rather than AB plasma significantly increased the survival rates. The FDA has cleared ASC930 for a phase 2 trial for Grade II-IV SR-aGVHD, to confirm the manufacturability, safety and efficacy data already demonstrated in academic trials of DSCs.

The future of allogeneic cell therapies in SR-aGVHD will be a crucial milestone, not only for this indication, also for the demonstration that placenta-derived DSCs can exert a potent immunoregulatory role in patients with life-threatening conditions and potentially with autoimmune diseases. With the promise of these therapies also comes the expansion of therapeutic indications, determination of relevant endpoints, both clinical and surrogate biomarkers, as well as inclusion/exclusion criteria to target the right patient population in bespoke clinical trials. Among the most difficult challenges for allogeneic cell therapies is ensuring reproducibility and large-scale manufacturability. Scaling up these therapies is time-intensive and costly with significant obstacles halting or slowing down their path to clinical use. This potential bottleneck in the availability of clinical materials requires the collaborative efforts of academia and industry to avoid supply chain disruptions and ensure accelerated progress in the cell therapy space. Future automation solutions may alleviate some of these issues, including reducing human error, enhancing reproducibility, and achieving standardization. Automation holds the potential to assist with the critical quality control necessary for cell therapy. Interest from investors and manufacturers is increasing with successful clinical trials and hopefully, will assist successful therapies in reaching the production and manufacturing phases.

The advantages of using DSCs over MSCs make them an attractive alternative to inflammatory-related diseases. Although still under investigation, some efficacy and safety limitations seen with MSC-based therapy have been found to be circumvented when using DSCs instead. More research is also required to identify the long-term effects of DSC-based therapies beyond one-to-five-year survival data for various clinical indications. We also need to determine the potential of repeated treatment cycles, compared with a potentially safer ‘one-and-done’ single infusion, given the pharmacokinetic and pharmacodynamic profile of the ‘hit-and-run’ nature of DSCs.

The progress already achieved with DSC-based therapies in pre- and clinical development programs for aGVHD so far is paving the way toward the approval and mainstream use of these therapies in the clinic. DSC-based treatments will reach full-scale production and manufacturing phases over the next decade. The present indications for most cell therapies include diseases with few to no current treatments, high mortality rates, and substantial threats to the quality of life. It will also be interesting to see the use for cell therapy treatments broaden in the near future, which is bound to occur with the safety and efficacy data reported by current clinical trials.

## Author contributions

The authors confirm substantial contributions and revisions of this manuscript and approve it for publication.

## References

[1] Granot N Storb R. History of hematopoietic cell transplantation: challenges and progress. Haematologica (2020) 105(12):2716–29. doi: 10.3324/haematol.2019.245688 PMC7716373 33054108

[2] Passweg JR Baldomero H Chabannon C Basak GW de la Cámara R Corbacioglu S. Hematopoietic cell transplantation and cellular therapy survey of the EBMT: monitoring of activities and trends over 30 years. Bone Marrow Transplantation (2021) 56(7):1651–64. doi: 10.1038/s41409-021-01227-8 PMC8263343 33623153

[3] Phelan R Arora M Chen M. Current use and outcome of hematopoietic stem cell transplantation: CIBMTR US summary slides. (2020). doi: 10.1016/j.bbmt.2020.04.013

[4] Kanate AS Majhail NS Savani BN Bredeson C Champlin RE Crawford S. Indications for hematopoietic cell transplantation and immune effector cell therapy: guidelines from the american society for transplantation and cellular therapy. Biol Blood Marrow Transplant (2020) 26(7):1247–56. doi: 10.1016/j.bbmt.2020.03.002 32165328

[5] D'Souza A Fretham C Lee SJ Arora M Brunner J Chhabra S. Current use of and trends in hematopoietic cell transplantation in the United States. Biol Blood Marrow Transplant (2020) 26(8):e177–e82. doi: 10.1016/j.bbmt.2020.04.013 PMC7404814 32438042

[6] Ferrara JL Levine JE Reddy P Holler E. Graft-versus-host disease. Lancet (2009) 373(9674):1550–61. doi: 10.1016/S0140-6736(09)60237-3 PMC2735047 19282026

[7] Zeiser R Negrin RS. Introduction to a review series on chronic GVHD: from pathogenic B-cell receptor signaling to novel therapeutic targets. Blood (2017) 129(1):1–2. doi: 10.1182/blood-2016-10-735696 27821507

[8] Harris AC Young R Devine S Hogan WJ Ayuk F Bunworasate U. International, multicenter standardization of acute graft-versus-host disease clinical data collection: A report from the mount sinai acute GVHD international consortium. Biol Blood Marrow Transplant (2016) 22(1):4–10. doi: 10.1016/j.bbmt.2015.09.001 26386318 PMC4706482

[9] Jagasia MH Greinix HT Arora M Williams KM Wolff D Cowen EW. National institutes of health consensus development project on criteria for clinical trials in chronic graft-versus-host disease: I. The 2014 diagnosis and staging working group report. Biol Blood Marrow Transplant (2015) 21(3):389–401.e1. doi: 10.1016/j.bbmt.2014.12.001 25529383 PMC4329079

[10] Schoemans HM Lee SJ Ferrara JL Wolff D Levine JE Schultz KR. EBMT-NIH-CIBMTR Task Force position statement on standardized terminology & guidance for graft-versus-host disease assessment. Bone Marrow Transplant (2018) 53(11):1401–15. doi: 10.1038/s41409-018-0204-7 PMC6786777 29872128

[11] Jamil MO Mineishi S. State-of-the-art acute and chronic GVHD treatment. Int J Hematol (2015) 101(5):452–66. doi: 10.1007/s12185-015-1785-1 25864189

[12] Ramachandran V Kolli SS Strowd LC. Review of graft-versus-host disease. Dermatologic Clinics (2019) 37(4):569–82. doi: 10.1016/j.det.2019.05.014 31466596

[13] Jagasia M Arora M Flowers ME Chao NJ McCarthy PL Cutler CS. Risk factors for acute GVHD and survival after hematopoietic cell transplantation. Blood (2012) 119(1):296–307. doi: 10.1182/blood-2011-06-364265 22010102 PMC3251233

[14] Zeiser R Blazar BR. Acute graft-versus-host disease — Biologic process, prevention, and therapy. New Engl J Med (2017) 377(22):2167–79. doi: 10.1056/NEJMra1609337 PMC6034180 29171820

[15] Mathew NR Vinnakota JM Apostolova P Erny D Hamarsheh S Andrieux G. Graft-versus-host disease of the CNS is mediated by TNF upregulation in microglia. J Clin Invest (2020) 130(3):1315–29. doi: 10.1172/JCI130272 PMC7269577 31846439

[16] Zeiser R Teshima T. Nonclassical manifestations of acute GVHD. Blood (2021) 138(22):2165–72. doi: 10.1182/blood.2021012431 34482399

[17] Bhatt VR Wang T Chen K Kitko CL MacMillan ML Pidala JA. Chronic graft-versus-host disease, nonrelapse mortality, and disease relapse in older versus younger adults undergoing matched allogeneic peripheral blood hematopoietic cell transplantation: A center for international blood and marrow transplant research analysis. Transplant Cell Ther (2022) 28(1):34–42. doi: 10.1016/j.jtct.2021.10.002 34637965 PMC8792177

[18] DeFilipp Z Onstad L Arai S Arora M Cutler C Flowers ME. Non-relapse mortality among patients diagnosed with chronic graft-versus-host disease: an updated analysis from the chronic GVHD consortium. 2021 TCT| Transplant Cell Ther Meetings ASTCT CIBMTR (2021). doi: 10.1016/S2666-6367(21)00109-3

[19] Bachier CR Aggarwal SK Hennegan K Milgroom A Francis K Dehipawala S. Epidemiology and treatment of chronic graft-versus-host disease post-allogeneic hematopoietic cell transplantation: A US claims analysis. Transplant Cell Ther (2021) 27(6):504. e1–. e6. doi: 10.1016/j.jtct.2020.12.027 34158154

[20] Pavletic SZ Martin PJ Schultz KR Lee SJ. The future of chronic graft-versus-host disease: introduction to the 2020 national institutes of health consensus development project reports. Transplant Cell Ther (2021) 27(6):448–51. doi: 10.1016/j.jtct.2021.02.034 33785366

[21] Arai S Arora M Wang T Spellman SR He W Couriel DR. Increasing incidence of chronic graft-versus-host disease in allogeneic transplantation: A report from the center for international blood and marrow transplant research. Biol Blood Marrow Transplantat (2015) 21(2):266–74. doi: 10.1016/j.bbmt.2014.10.021 PMC4326247 25445023

[22] Lee SJ. Classification systems for chronic graft-versus-host disease. Blood (2017) 129(1):30–7. doi: 10.1182/blood-2016-07-686642 PMC5216262 27821503

[23] Zeiser R Blazar BR. Pathophysiology of chronic graft-versus-host disease and therapeutic targets. N Engl J Med (2017) 377(26):2565–79. doi: 10.1056/NEJMra1703472 29281578

[24] Hamilton BK. Updates in chronic graft-versus-host disease. Hematology (2021) 2021(1):648–54. doi: 10.1182/hematology.2021000301 PMC8791178 34889364

[25] Ferrara JL Cooke KR Teshima T. The pathophysiology of acute graft-versus-host disease. Int J Hematol (2003) 78(3):181–7. doi: 10.1007/BF02983793 14604275

[26] Reddy P. Pathophysiology of acute graft-versus-host disease. Hematol Oncol (2003) 21(4):149–61. doi: 10.1002/hon.716 14735553

[27] Weiden PL Sullivan KM Flournoy N Storb R Thomas ED Seattle Marrow Transplant T. Antileukemic effect of chronic graft-versus-host disease: contribution to improved survival after allogeneic marrow transplantation. N Engl J Med (1981) 304(25):1529–33. doi: 10.1056/NEJM198106183042507 7015133

[28] Horowitz MM Gale RP Sondel PM Goldman JM Kersey J Kolb HJ. Graft-versus-leukemia reactions after bone marrow transplantation. Blood (1990) 75(3):555–62. doi: 10.1182/blood.V75.3.555.555 2297567

[29] Ringden O Pavletic SZ Anasetti C Barrett AJ Wang T Wang D. The graft-versus-leukemia effect using matched unrelated donors is not superior to HLA-identical siblings for hematopoietic stem cell transplantation. Blood (2009) 113(13):3110–8. doi: 10.1182/blood-2008-07-163212 PMC2662650 19059878

[30] Wilhelm K Ganesan J Muller T Durr C Grimm M Beilhack A. Graft-versus-host disease is enhanced by extracellular ATP activating P2X7R. Nat Med (2010) 16(12):1434–8. doi: 10.1038/nm.2242 21102458

[31] Nassereddine S Rafei H Elbahesh E Tabbara I. Acute graft versus host disease: A comprehensive review. Anticancer Res (2017) 37(4):1547–55. doi: 10.21873/anticanres.11483 28373413

[32] Ghimire S Weber D Mavin E Wang XN Dickinson AM Holler E. Pathophysiology of gvHD and other HSCT-related major complications. Front Immunol (2017) 8:79. doi: 10.3389/fimmu.2017.00079 28373870 PMC5357769

[33] Sun Y Tawara I Toubai T Reddy P. Pathophysiology of acute graft-versus-host disease: recent advances. Transl Res (2007) 150(4):197–214. doi: 10.1016/j.trsl.2007.06.003 17900507 PMC2084257

[34] Teshima T Hill GR. The pathophysiology and treatment of graft-versus-host disease: lessons learnt from animal models. Front Immunol (2021) 12:715424. doi: 10.3389/fimmu.2021.715424 34489966 PMC8417310

[35] Cooke KR Luznik L Sarantopoulos S Hakim FT Jagasia M Fowler DH. The biology of chronic graft-versus-host disease: A task force report from the national institutes of health consensus development project on criteria for clinical trials in chronic graft-versus-host disease. Biol Blood Marrow Transplant (2017) 23(2):211–34. doi: 10.1016/j.bbmt.2016.09.023 PMC6020045 27713092

[36] MacDonald KP Hill GR Blazar BR. Chronic graft-versus-host disease: biological insights from preclinical and clinical studies. Blood (2017) 129(1):13–21. doi: 10.1182/blood-2016-06-686618 27821504 PMC5216261

[37] Blazar BR Murphy WJ Abedi M. Advances in graft-versus-host disease biology and therapy. Nat Rev Immunol (2012) 12(6):443–58. doi: 10.1038/nri3212 PMC3552454 22576252

[38] Zeiser R Lee SJ. Three US Food and Drug Administration-approved therapies for chronic GVHD. Blood (2022) 139(11):1642–5. doi: 10.1182/blood.2021014448 PMC8931512 35081254

[39] Naserian S Leclerc M Shamdani S Uzan G. Current preventions and treatments of aGVHD: from pharmacological prophylaxis to innovative therapies. Front Immunol (2020) 11:607030. doi: 10.3389/fimmu.2020.607030 33391276 PMC7773902

[40] Hamilton BK. Current approaches to prevent and treat GVHD after allogeneic stem cell transplantation. Hematology (2018) 2018(1):228–35. doi: 10.1182/asheducation-2018.1.228 PMC6246030 30504315

[41] Gooptu M Antin JH. GVHD prophylaxis 2020. Front Immunol (2021) 12:605726. doi: 10.3389/fimmu.2021.605726 33897681 PMC8059368

[42] ORENCIA. PRESCRIBING INFORMATION. Bristol-Myers Squibb (2021).

[43] Penack O Marchetti M Ruutu T Aljurf M Bacigalupo A Bonifazi F. Prophylaxis and management of graft versus host disease after stem-cell transplantation for haematological Malignancies: updated consensus recommendations of the European Society for Blood and Marrow Transplantation. Lancet Haematol (2020) 7(2):e157–e67. doi: 10.1016/S2352-3026(19)30256-X 32004485

[44] Martin PJ Rizzo JD Wingard JR Ballen K Curtin PT Cutler C. First- and second-line systemic treatment of acute graft-versus-host disease: recommendations of the American Society of Blood and Marrow Transplantation. Biol Blood Marrow Transplant (2012) 18(8):1150–63. doi: 10.1016/j.bbmt.2012.04.005 PMC3404151 22510384

[45] Ruutu T Gratwohl A de Witte T Afanasyev B Apperley J Bacigalupo A. Prophylaxis and treatment of GVHD: EBMT-ELN working group recommendations for a standardized practice. Bone Marrow Transplant (2014) 49(2):168–73. doi: 10.1038/bmt.2013.107 23892326

[46] Mielcarek M Furlong T Storer BE Green ML McDonald GB Carpenter PA. Effectiveness and safety of lower dose prednisone for initial treatment of acute graft-versus-host disease: a randomized controlled trial. Haematologica (2015) 100(6):842–8. doi: 10.3324/haematol.2014.118471 PMC4450631 25682602

[47] Malard F Huang XJ Sim JPY. Treatment and unmet needs in steroid-refractory acute graft-versus-host disease. Leukemia (2020) 34(5):1229–40. doi: 10.1038/s41375-020-0804-2 PMC7192843 32242050

[48] Inamoto Y Martin PJ Storer BE Mielcarek M Storb RF Carpenter PA. Response endpoints and failure-free survival after initial treatment for acute graft-versus-host disease. Haematologica (2014) 99(2):385–91. doi: 10.3324/haematol.2013.093062 PMC3912972 24056814

[49] Xhaard A Rocha V Bueno B de Latour RP Lenglet J Petropoulou A. Steroid-refractory acute GVHD: lack of long-term improved survival using new generation anticytokine treatment. Biol Blood Marrow Transplant. (2012) 18(3):406–13. doi: 10.1016/j.bbmt.2011.06.012 21736868

[50] Martin PJ. How I treat steroid-refractory acute graft-versus-host disease. Blood (2020) 135(19):1630–8. doi: 10.1182/blood.2019000960 32202630

[51] Hill L Alousi A Kebriaei P Mehta R Rezvani K Shpall E. New and emerging therapies for acute and chronic graft versus host disease. Ther Adv hematol (2018) 9(1):21–46. doi: 10.1177/2040620717741860 29317998 PMC5753923

[52] Dignan FL Clark A Amrolia P Cornish J Jackson G Mahendra P. Diagnosis and management of acute graft-versus-host disease. Br J Haematol (2012) 158(1):30–45. doi: 10.1111/j.1365-2141.2012.09129.x 22533831

[53] Turner L. The American stem cell sell in 2021: U.S. businesses selling unlicensed and unproven stem cell interventions. Cell Stem Cell (2021) 28(11):1891–5. doi: 10.1016/j.stem.2021.10.008 34739831

[54] Nassar A Elgohary G Elhassan T Nurgat Z Mohamed SY Aljurf M. Methotrexate for the treatment of graft-versus-host disease after allogeneic hematopoietic stem cell transplantation. J Transplantat (2014) 2014:980301. doi: 10.1155/2014/980301 PMC4227326 25405023

[55] Yalniz FF Murad MH Lee SJ Pavletic SZ Khera N Shah ND. Steroid refractory chronic graft-versus-host disease: cost-effectiveness analysis. Biol Blood Marrow Transplant (2018) 24(9):1920–7. doi: 10.1016/j.bbmt.2018.03.008 PMC8058632 29550629

[56] Drexler B Buser A Infanti L Stehle G Halter J Holbro A. Extracorporeal photopheresis in graft-versus-host disease. Transfusion Med Hemother (2020) 47(3):214–25. doi: 10.1159/000508169 PMC7315199 32595426

[57] Schub N Günther A Schrauder A Claviez A Ehlert C Gramatzki M. Therapy of steroid-refractory acute GVHD with CD52 antibody alemtuzumab is effective. Bone Marrow Transplant (2011) 46:143–7. doi: 10.1038/bmt.2010.68 20348971

[58] Schabert VF Watson C Joseph GJ Iversen P Burudpakdee C Harrison DJ. Costs of tumor necrosis factor blockers per treated patient using real-world drug data in a managed care population. J Manag Care Pharm (2013) 19(8):621–30. doi: 10.18553/jmcp.2013.19.8.621 PMC10438048 24074008

[59] Adkins BD Kneib J Plummer WD Dupont WD Booth GS. Extracorporeal photopheresis chargemasters show haphazard billing practices. Transfusion (2021) 61:2844–2848. doi: 10.1111/trf.16602 34297353

[60] Meunier M Bulabois CE Thiebaut-Bertrand A Itzykson R Carre M Carras S. Alemtuzumab for severe steroid-refractory gastrointestinal acute graft-versus-host disease. Biol Blood Marrow Transplant (2014) 20(9):1451–4. doi: 10.1016/j.bbmt.2014.05.031 24910381

[61] Gómez-Almaguer D Ruiz-Argüelles GJ del Carmen Tarín-Arzaga L González-Llano O Gutiérrez-Aguirre H Cantú-Rodríguez O. Alemtuzumab for the treatment of steroid-refractory acute graft-versus-host disease. Biol Blood Marrow Transplant (2008) 14(1):10–5. doi: 10.1016/j.bbmt.2007.08.052 18158956

[62] Martínez C Solano C Ferrá C Sampol A Valcárcel D Pérez-Simón JA. Alemtuzumab as treatment of steroid-refractory acute graft-versus-host disease: results of a phase II study. Biol Blood Marrow Transplant (2009) 15(5):639–42. doi: 10.1016/j.bbmt.2009.01.014 19361757

[63] Holtan SG Ustun C Hoeschen A Feola J Cao Q Gandhi P. Phase 2 results of urinary-derived human chorionic gonadotropin/epidermal growth factor as treatment for life-threatening acute gvhd. Blood (2021) 138(Supplement 1):261. doi: 10.1182/blood-2021-145008

[64] Holtan SG Hoeschen A Cao Q Arora M Bachanova V Brunstein CG. Facilitating resolution of life-threatening acute graft-versus-host disease by supplementation of human chorionic gonadotropin and epidermal growth factor (Pregnyl): A phase I study. Blood (2018) 132(Supplement 1):71. doi: 10.1182/blood-2018-99-113564

[65] Holtan SG Hoeschen AL Cao Q Arora M Bachanova V Brunstein CG. Facilitating resolution of life-threatening acute GVHD with human chorionic gonadotropin and epidermal growth factor. Blood Advances (2020) 4(7):1284–95. doi: 10.1182/bloodadvances.2019001259 PMC7160256 32236525

[66] Mancusi A Piccinelli S Velardi A Pierini A. The effect of TNF-α on regulatory T cell function in graft-versus-host disease. Front Immunol (2018) 9:356. doi: 10.3389/fimmu.2018.00356 29541073 PMC5835761

[67] Park JH Lee HJ Kim SR Song GW Lee SK Park SY. Etanercept for steroid-refractory acute graft versus host disease following allogeneic hematopoietic stem cell transplantation. Korean J Internal Med (2014) 29(5):630–6. doi: 10.3904/kjim.2014.29.5.630 PMC4164727 25228839

[68] Furlong T Martin P Flowers M Carnevale-Schianca F Yatscoff R Chauncey T. Therapy with mycophenolate mofetil for refractory acute and chronic GVHD. Bone Marrow Transplant (2009) 44:739–48. doi: 10.1038/bmt.2009.76 PMC2791193 19377515

[69] James A Mannon RB. The cost of transplant immunosuppressant therapy: is this sustainable? Curr Transplant Rep (2015) 2(2):113–21. doi: 10.1007/s40472-015-0052-y PMC4520417 26236578

[70] Braun LM Zeiser R. Kinase inhibition as treatment for acute and chronic graft-versus-host disease. Front Immunol (2021) 12. doi: 10.3389/fimmu.2021.760199 PMC8635802 34868001

[71] Pidala J Hamadani M Dawson P Martens M Alousi AM Jagasia M. Randomized multicenter trial of sirolimus vs prednisone as initial therapy for standard-risk acute GVHD: the BMT CTN 1501 trial. Blood (2020) 135(2):97–107. doi: 10.1182/blood.2019003125 31738834 PMC6952830

[72] Shen M Li J Zhang X Xu L Wang Y Liu K. Meta-analysis of interleukin-2 receptor antagonists as the treatment for steroid-refractory acute graft-versus-host disease. Front Immunol (2021) 12:749266. doi: 10.3389/fimmu.2021.749266 34621279 PMC8490710

[73] Baron F Mohty M Blaise D Socié G Labopin M Esteve J. Anti-thymocyte globulin as graft-versus-host disease prevention in the setting of allogeneic peripheral blood stem cell transplantation: A review from the acute leukemia working party of the european society for blood and marrow transplantation. Haematologica (2017) 102(2):224–34. doi: 10.3324/haematol.2016.148510 PMC5286931 27927772

[74] Przepiorka D Luo L Subramaniam S Qiu J Gudi R Cunningham LC. FDA approval summary: ruxolitinib for treatment of steroid-refractory acute graft-versus-host disease. Oncol (2020) 25(2):e328–e34. doi: 10.1634/theoncologist.2019-0627 PMC7011641 32043777

[75] González Vicent M Molina B González de Pablo J Castillo A Díaz MÁ. Ruxolitinib treatment for steroid refractory acute and chronic graft vs host disease in children: Clinical and immunological results. Am J Hematol (2019) 94(3):319–26. doi: 10.1002/ajh.25376 30536806

[76] Jagasia M Perales MA Schroeder MA Ali H Shah NN Chen YB. Ruxolitinib for the treatment of steroid-refractory acute GVHD (REACH1): a multicenter, open-label phase 2 trial. Blood (2020) 135(20):1739–49. doi: 10.1182/blood.2020004823 PMC7229262 32160294

[77] Zeiser R von Bubnoff N Butler J Mohty M Niederwieser D Or R. Ruxolitinib for glucocorticoid-refractory acute graft-versus-host disease. N Engl J Med (2020) 382(19):1800–10. doi: 10.1056/NEJMoa1917635 32320566

[78] Flowers ME Martin PJ. How we treat chronic graft-versus-host disease. Blood (2015) 125(4):606–15. doi: 10.1182/blood-2014-08-551994 PMC4304105 25398933

[79] Dignan FL Amrolia P Clark A Cornish J Jackson G Mahendra P. Diagnosis and management of chronic graft-versus-host disease. Br J haematol (2012) 158(1):46–61. doi: 10.1111/j.1365-2141.2012.09128.x 22533811

[80] Miklos D Cutler CS Arora M Waller EK Jagasia M Pusic I. Ibrutinib for chronic graft-versus-host disease after failure of prior therapy. Blood (2017) 130(21):2243–50. doi: 10.1182/blood-2017-07-793786 PMC6033048 28924018

[81] Waller EK Miklos D Cutler C Arora M Jagasia MH Pusic I. Ibrutinib for chronic graft-versus-host disease after failure of prior therapy: 1-year update of a phase 1b/2 study. Biol Blood Marrow Transplant (2019) 25(10):2002–7. doi: 10.1016/j.bbmt.2019.06.023 31260802

[82] Cutler C Lee SJ Arai S Rotta M Zoghi B Lazaryan A. Belumosudil for chronic graft-versus-host disease after 2 or more prior lines of therapy: the ROCKstar Study. Blood (2021) 138(22):2278–89. doi: 10.1182/blood.2021012021 PMC8641099 34265047

[83] Bachier CR Skaar JR Dehipawala S Miao B Ieyoub J Taitel H. Budget impact analysis of belumosudil for chronic graft-versus-host disease treatment in the United States. J Med Econ (2022) 25(1):857–63. doi: 10.1080/13696998.2022.2087408 35674411

[84] Zeiser R Polverelli N Ram R Hashmi SK Chakraverty R Middeke JM. Ruxolitinib for glucocorticoid-refractory chronic graft-versus-host disease. N Engl J Med (2021) 385(3):228–38. doi: 10.1056/NEJMoa2033122 34260836

[85] Kitko CL Pidala J Schoemans HM Lawitschka A Flowers ME Cowen EW. National institutes of health consensus development project on criteria for clinical trials in chronic graft-versus-host disease: IIa. The 2020 clinical implementation and early diagnosis working group report. Transplant Cell Ther (2021) 27(7):545–57. doi: 10.1016/j.jtct.2021.03.033 PMC8803210 33839317

[86] Pidala J Kitko C Lee SJ Carpenter P Cuvelier GDE Holtan S. National institutes of health consensus development project on criteria for clinical trials in chronic graft-versus-host disease: IIb. The 2020 preemptive therapy working group report. Transplant Cell Ther (2021) 27(8):632–41. doi: 10.1016/j.jtct.2021.03.029 PMC8934187 33836313

[87] Alabdaljabar MS Aslam HM Veeraballi S Faizee FA Husain BH Iqbal SM. Restoration of the original inhabitants: A systematic review on fecal microbiota transplantation for graft-versus-host disease. Cureus (2022) 14(4):e23873. doi: 10.7759/cureus.23873 35530905 PMC9076056

[88] van Lier YF Davids M Haverkate NJE de Groot P Donker ML Meijer E. Donor fecal microbiota transplantation ameliorates intestinal graft-versus-host disease in allogeneic hematopoietic cell transplant recipients. Sci Trans Med (2020) 12:eaaz8926. doi: 10.1126/scitranslmed.aaz8926 32801142

[89] Hefazi M Bolivar-Wagers S Blazar BR. Regulatory T cell therapy of graft-versus-host disease: advances and challenges. Int J Mol Sci (2021) 22(18). doi: 10.3390/ijms22189676 PMC8469916 34575843

[90] Guo W-w Su X-h Wang M-y Han M-z Feng X-m Jiang E-l. Regulatory T cells in GVHD therapy. Front Immunol (2021) 12. doi: 10.3389/fimmu.2021.697854 PMC8250864 34220860

[91] Ullah I Subbarao RB Rho GJ. Human mesenchymal stem cells - current trends and future prospective. Biosci Rep (2015) 35(2). doi: 10.1042/BSR20150025 PMC4413017 25797907

[92] Keating A. Mesenchymal stromal cells. Curr Opin Hematol (2006) 13(6):419–25. doi: 10.1097/01.moh.0000245697.54887.6f PMC3365862 17053453

[93] Murray IR Péault B. Q&A: Mesenchymal stem cells - where do they come from and is it important? BMC Biol (2015) 13:99. doi: 10.1186/s12915-015-0212-7 26596888 PMC4656175

[94] Andrzejewska A Lukomska B Janowski M. Concise review: mesenchymal stem cells: from roots to boost. Stem Cells (2019) 37(7):855–64. doi: 10.1002/stem.3016 PMC6658105 30977255

[95] Hass R Kasper C Böhm S Jacobs R. Different populations and sources of human mesenchymal stem cells (MSC): A comparison of adult and neonatal tissue-derived MSC. Cell Commun Signal (2011) 9:12. doi: 10.1186/1478-811X-9-12 21569606 PMC3117820

[96] Galipeau J Sensébé L. Mesenchymal stromal cells: clinical challenges and therapeutic opportunities. Cell Stem Cell (2018) 22(6):824–33. doi: 10.1016/j.stem.2018.05.004 PMC6434696 29859173

[97] Rendra E Scaccia E Bieback K. Recent advances in understanding mesenchymal stromal cells. F1000Res (2020) 9. doi: 10.12688/f1000research.21862.1 PMC7047922 32148780

[98] Elgaz S Kuçi Z Kuçi S Bönig H Bader P. Clinical use of mesenchymal stromal cells in the treatment of acute graft-versus-host disease. Transfusion Med Hemother (2019) 46(1):27–34. doi: 10.1159/000496809 PMC6558336 31244579

[99] Li T Luo C Zhang J Wei L Sun W Xie Q. Efficacy and safety of mesenchymal stem cells co-infusion in allogeneic hematopoietic stem cell transplantation: a systematic review and meta-analysis. Stem Cell Res Ther (2021) 12(1):246. doi: 10.1186/s13287-020-02064-0 33879242 PMC8056684

[100] Thompson M Mei SHJ Wolfe D Champagne J Fergusson D Stewart DJ. Cell therapy with intravascular administration of mesenchymal stromal cells continues to appear safe: An updated systematic review and meta-analysis. EClinicalMedicine (2020) 19:100249. doi: 10.1016/j.eclinm.2019.100249 31989101 PMC6970160

[101] Spees JL Lee RH Gregory CA. Mechanisms of mesenchymal stem/stromal cell function. Stem Cell Res Ther (2016) 7(1):125. doi: 10.1186/s13287-016-0363-7 27581859 PMC5007684

[102] Harrell CR Jovicic N Djonov V Arsenijevic N Volarevic V. Mesenchymal stem cell-derived exosomes and other extracellular vesicles as new remedies in the therapy of inflammatory diseases. Cells (2019) 8(12). doi: 10.3390/cells8121605 PMC6952783 31835680

[103] Weiss ARR Dahlke MH. Immunomodulation by mesenchymal stem cells (MSCs): mechanisms of action of living, apoptotic, and dead MSCs. Front Immunol (2019) 10. doi: 10.3389/fimmu.2019.01191 PMC6557979 31214172

[104] Shen Z Huang W Liu J Tian J Wang S Rui K. Effects of mesenchymal stem cell-derived exosomes on autoimmune diseases. Front Immunol (2021) 12:749192. doi: 10.3389/fimmu.2021.749192 34646275 PMC8503317

[105] Burnham AJ Daley-Bauer LP Horwitz EM. Mesenchymal stromal cells in hematopoietic cell transplantation. Blood Adv (2020) 4(22):5877–87. doi: 10.1182/bloodadvances.2020002646 PMC7686890 33232479

[106] Uccelli A de Rosbo NK. The immunomodulatory function of mesenchymal stem cells: mode of action and pathways. Ann N Y Acad Sci (2015) 1351:114–26. doi: 10.1111/nyas.12815 26152292

[107] Voermans C Hazenberg MD. Cellular therapies for graft-versus-host disease: a tale of tissue repair and tolerance. Blood (2020) 136(4):410–7. doi: 10.1182/blood.2019000951 32525970

[108] Godoy JAP Paiva RMA Souza AM Kondo AT Kutner JM Okamoto OK. Clinical translation of mesenchymal stromal cell therapy for graft versus host disease. Front Cell Dev Biol (2019) 7:255. doi: 10.3389/fcell.2019.00255 31824942 PMC6881464

[109] Wu X Jiang J Gu Z Zhang J Chen Y Liu X. Mesenchymal stromal cell therapies: immunomodulatory properties and clinical progress. Stem Cell Res Ther (2020) 11(1):345. doi: 10.1186/s13287-020-01855-9 32771052 PMC7414268

[110] Le Blanc K Rasmusson I Sundberg B Götherström C Hassan M Uzunel M. Treatment of severe acute graft-versus-host disease with third party haploidentical mesenchymal stem cells. Lancet (2004) 363(9419):1439–41. doi: 10.1016/S0140-6736(04)16104-7 15121408

[111] Le Blanc K Frassoni F Ball L Locatelli F Roelofs H Lewis I. Mesenchymal stem cells for treatment of steroid-resistant, severe, acute graft-versus-host disease: a phase II study. Lancet (2008) 371(9624):1579–86. doi: 10.1016/S0140-6736(08)60690-X 18468541

[112] Zhao L Chen S Yang P Cao H Li L. The role of mesenchymal stem cells in hematopoietic stem cell transplantation: prevention and treatment of graft-versus-host disease. Stem Cell Res Ther (2019) 10(1):182. doi: 10.1186/s13287-019-1287-9 31227011 PMC6588914

[113] Murata M Teshima T. Treatment of steroid-refractory acute graft-versus-host disease using commercial mesenchymal stem cell products. Front Immunol (2021) 12:724380. doi: 10.3389/fimmu.2021.724380 34489977 PMC8417106

[114] Fisher SA Cutler A Doree C Brunskill SJ Stanworth SJ Navarrete C. Mesenchymal stromal cells as treatment or prophylaxis for acute or chronic graft-versus-host disease in haematopoietic stem cell transplant (HSCT) recipients with a haematological condition. Cochrane Database Syst Rev (2019) 1(1):Cd009768. doi: 10.1002/14651858.CD009768.pub2 30697701 PMC6353308

[115] Introna M Golay J. Tolerance to bone marrow transplantation: do mesenchymal stromal cells still have a future for acute or chronic gvHD? Front Immunol (2020) 11:609063. doi: 10.3389/fimmu.2020.609063 33362797 PMC7759493

[116] Cheung TS Bertolino GM Giacomini C Bornhäuser M Dazzi F Galleu A. Mesenchymal stromal cells for graft versus host disease: mechanism-based biomarkers. Front Immunol (2020) 11. doi: 10.3389/fimmu.2020.01338 PMC7330053 32670295

[117] Trento C Bernardo ME Nagler A Kuçi S Bornhäuser M Köhl U. Manufacturing mesenchymal stromal cells for the treatment of graft-versus-host disease: A survey among centers affiliated with the european society for blood and marrow transplantation. Biol Blood Marrow Transplant (2018) 24(11):2365–70. doi: 10.1016/j.bbmt.2018.07.015 PMC6299357 30031938

[118] Wright A Arthaud-Day ML Weiss ML. Therapeutic use of mesenchymal stromal cells: the need for inclusive characterization guidelines to accommodate all tissue sources and species. Front Cell Dev Biol (2021) 9. doi: 10.3389/fcell.2021.632717 PMC7921162 33665190

[119] Mizukami A Swiech K. Mesenchymal stromal cells: from discovery to manufacturing and commercialization. Stem Cells Int (2018) 2018:4083921. doi: 10.1155/2018/4083921 30057622 PMC6051015

[120] Kebriaei P Isola L Bahceci E Holland K Rowley S McGuirk J. Adult human mesenchymal stem cells added to corticosteroid therapy for the treatment of acute graft-versus-host disease. Biol Blood Marrow Transplant (2009) 15(7):804–11. doi: 10.1016/j.bbmt.2008.03.012 19539211

[121] Kebriaei P Hayes J Daly A Uberti J Marks DI Soiffer R. A phase 3 randomized study of remestemcel-L versus placebo added to second-line therapy in patients with steroid-refractory acute graft-versus-host disease. Biol Blood Marrow Transplant (2020) 26(5):835–44. doi: 10.1016/j.bbmt.2019.08.029 PMC7060124 31505228

[122] Muroi K Miyamura K Ohashi K Murata M Eto T Kobayashi N. Unrelated allogeneic bone marrow-derived mesenchymal stem cells for steroid-refractory acute graft-versus-host disease: a phase I/II study. Int J Hematol (2013) 98(2):206–13. doi: 10.1007/s12185-013-1399-4 23860964

[123] Muroi K Miyamura K Okada M Yamashita T Murata M Ishikawa T. Bone marrow-derived mesenchymal stem cells (JR-031) for steroid-refractory grade III or IV acute graft-versus-host disease: a phase II/III study. Int J Hematol (2016) 103(2):243–50. doi: 10.1007/s12185-015-1915-9 26608364

[124] Kurtzberg J Abdel-Azim H Carpenter P Chaudhury S Horn B Mahadeo K. A phase 3, single-arm, prospective study of remestemcel-L, ex vivo culture-expanded adult human mesenchymal stromal cells for the treatment of pediatric patients who failed to respond to steroid treatment for acute graft-versus-host disease. Biol Blood Marrow Transplant (2020) 26(5):845–54. doi: 10.1016/j.bbmt.2020.01.018 PMC8322819 32018062

[125] Kurtzberg J Prockop S Chaudhury S Horn B Nemecek E Prasad V. Study 275: updated expanded access program for remestemcel-L in steroid-refractory acute graft-versus-host disease in children. Biol Blood Marrow Transplant (2020) 26(5):855–64. doi: 10.1016/j.bbmt.2020.01.026 PMC8292970 32044400

[126] Daly A. Remestemcel-L, the first cellular therapy product for the treatment of graft-versus-host disease. Drugs Today (Barc). (2012) 48(12):773–83. doi: 10.1358/dot.2012.48.12.1885866 23243634

[127] Murata M Terakura S Wake A Miyao K Ikegame K Uchida N. Off-the-shelf bone marrow-derived mesenchymal stem cell treatment for acute graft-versus-host disease: real-world evidence. Bone Marrow Transplant (2021) 56(10):2355–66. doi: 10.1038/s41409-021-01304-y 33976381

[128] Thäte C Woischwill C Brandenburg G Müller M Böhm S Baumgart J. Non-clinical assessment of safety, biodistribution and tumorigenicity of human mesenchymal stromal cells. Toxicol Rep (2021) 8:1960–9. doi: 10.1016/j.toxrep.2021.11.016 PMC8649581 34926173

[129] Kuçi Z Bönig H Kreyenberg H Bunos M Jauch A Janssen JW. Mesenchymal stromal cells from pooled mononuclear cells of multiple bone marrow donors as rescue therapy in pediatric severe steroid-refractory graft-versus-host disease: a multicenter survey. Haematologica (2016) 101(8):985–94. doi: 10.3324/haematol.2015.140368 PMC4967578 27175026

[130] Bader P Kuçi Z Bakhtiar S Basu O Bug G Dennis M. Effective treatment of steroid and therapy-refractory acute graft-versus-host disease with a novel mesenchymal stromal cell product (MSC-FFM). Bone Marrow Transplant. (2018) 53(7):852–62. doi: 10.1038/s41409-018-0102-z PMC6039391 29379171

[131] Adom D Rowan C Adeniyan T Yang J Paczesny S. Biomarkers for allogeneic HCT outcomes. Front Immunol (2020) 11:673. doi: 10.3389/fimmu.2020.00673 32373125 PMC7186420

[132] Kaviany S Kitko CL. The role of biomarkers in risk stratification, treatment and outcome in acute GVHD. Curr Opin Hematol (2021) 28(6):401–7. doi: 10.1097/MOH.0000000000000681 PMC10189358 34475350

[133] Nagasawa M. Biomarkers of graft-vs-host disease: Understanding and applications for the future. World J Transplant. (2021) 11(8):335–43. doi: 10.5500/wjt.v11.i8.335 PMC8371494 34447670

[134] Paczesny S Krijanovski OI Braun TM Choi SW Clouthier SG Kuick R. A biomarker panel for acute graft-versus-host disease. Blood (2009) 113(2):273–8. doi: 10.1182/blood-2008-07-167098 PMC2615645 18832652

[135] Levine JE Logan BR Wu J Alousi AM Bolaños-Meade J Ferrara JL. Acute graft-versus-host disease biomarkers measured during therapy can predict treatment outcomes: a Blood and Marrow Transplant Clinical Trials Network study. Blood (2012) 119(16):3854–60. doi: 10.1182/blood-2012-01-403063 PMC3335389 22383800

[136] Holtan SG Khera N Levine JE Chai X Storer B Liu HD. Late acute graft-versus-host disease: a prospective analysis of clinical outcomes and circulating angiogenic factors. Blood (2016) 128(19):2350–8. doi: 10.1182/blood-2015-09-669846 PMC5106113 27625357

[137] Luft T Conzelmann M Benner A Rieger M Hess M Strohhaecker U. Serum cytokeratin-18 fragments as quantitative markers of epithelial apoptosis in liver and intestinal graft-versus-host disease. Blood (2007) 110(13):4535–42. doi: 10.1182/blood-2006-10-049817 17702900

[138] Ferrara JL Harris AC Greenson JK Braun TM Holler E Teshima T. Regenerating islet-derived 3-alpha is a biomarker of gastrointestinal graft-versus-host disease. Blood (2011) 118(25):6702–8. doi: 10.1182/blood-2011-08-375006 PMC3242723 21979939

[139] Vander Lugt MT Braun TM Hanash S Ritz J Ho VT Antin JH. ST2 as a marker for risk of therapy-resistant graft-versus-host disease and death. N Engl J Med (2013) 369(6):529–39. doi: 10.1056/NEJMoa1213299 PMC3943357 23924003

[140] Solán L Kwon M Carbonell D Dorado N Balsalobre P Serrano D. ST2 and REG3α as predictive biomarkers after haploidentical stem cell transplantation using post-transplantation high-dose cyclophosphamide. Front Immunol (2019) 10:2338. doi: 10.3389/fimmu.2019.02338 31649665 PMC6794466

[141] Paczesny S Braun TM Levine JE Hogan J Crawford J Coffing B. Elafin is a biomarker of graft-versus-host disease of the skin. Sci Transl Med (2010) 2(13):13ra2. doi: 10.1126/scitranslmed.3000406 PMC2895410 20371463

[142] Srinagesh HK Özbek U Kapoor U Ayuk F Aziz M Ben-David K. The MAGIC algorithm probability is a validated response biomarker of treatment of acute graft-versus-host disease. Blood Adv (2019) 3(23):4034–42. doi: 10.1182/bloodadvances.2019000791 PMC6963240 31816061

[143] Major-Monfried H Renteria AS Pawarode A Reddy P Ayuk F Holler E. MAGIC biomarkers predict long-term outcomes for steroid-resistant acute GVHD. Blood (2018) 131(25):2846–55. doi: 10.1182/blood-2018-01-822957 PMC6014357 29545329

[144] Dander E Lucchini G Vinci P Introna M Masciocchi F Perseghin P. Mesenchymal stromal cells for the treatment of graft-versus-host disease: understanding the in vivo biological effect through patient immune monitoring. Leukemia (2012) 26(7):1681–4. doi: 10.1038/leu.2011.384 22289986

[145] von Bahr L Sundberg B Lönnies L Sander B Karbach H Hägglund H. Long-term complications, immunologic effects, and role of passage for outcome in mesenchymal stromal cell therapy. Biol Blood Marrow Transplant (2012) 18(4):557–64. doi: 10.1016/j.bbmt.2011.07.023 21820393

[146] Yin F Battiwalla M Ito S Feng X Chinian F Melenhorst JJ. Bone marrow mesenchymal stromal cells to treat tissue damage in allogeneic stem cell transplant recipients: correlation of biological markers with clinical responses. Stem Cells (2014) 32(5):1278–88. doi: 10.1002/stem.1638 PMC3991733 24452962

[147] Te Boome LC Mansilla C van der Wagen LE Lindemans CA Petersen EJ Spierings E. Biomarker profiling of steroid-resistant acute GVHD in patients after infusion of mesenchymal stromal cells. Leukemia (2015) 29(9):1839–46. doi: 10.1038/leu.2015.89 25836589

[148] Keto J Kaartinen T Salmenniemi U Castren J Partanen J Hanninen A. Immunomonitoring of MSC-treated gvHD patients reveals only moderate potential for response prediction but indicates treatment safety. Mol Ther Methods Clin Dev (2018) 9:109–18. doi: 10.1016/j.omtm.2018.02.001 PMC5834657 29516024

[149] ASH. Effect of remestemcel-L treatment in pediatric steroid refractory acute graft-versus-host disease: A biomarker substudy. Presented at Annu meeting Am Soc Hematol (ASH) (2020)

[150] Cheung TS Galleu A von Bonin M Bornhäuser M Dazzi F. Apoptotic mesenchymal stromal cells induce prostaglandin E2 in monocytes: implications for the monitoring of mesenchymal stromal cell activity. Haematologica (2019) 104(10):e438–e41. 10.3324/haematol.2018.214767 PMC6886441 30846505

[151] Galleu A Riffo-Vasquez Y Trento C Lomas C Dolcetti L Cheung TS. Apoptosis in mesenchymal stromal cells induces in vivo recipient-mediated immunomodulation. Sci Transl Med (2017) 9(416). doi: 10.1126/scitranslmed.aam7828 29141887

[152] Galleu Aea. Oral presentation, abstract S252, EHA. (2020)

[153] Trowsdale J Betz AG. Mother's little helpers: mechanisms of maternal-fetal tolerance. Nat Immunol (2006) 7(3):241–6. doi: 10.1038/ni1317 16482172

[154] Mori M Bogdan A Balassa T Csabai T Szekeres-Bartho J. The decidua-the maternal bed embracing the embryo-maintains the pregnancy. Semin Immunopathol (2016) 38(6):635–49. doi: 10.1007/s00281-016-0574-0 PMC5065593 27287066

[155] Ringdén O Erkers T Nava S Uzunel M Iwarsson E Conrad R. Fetal membrane cells for treatment of steroid-refractory acute graft-versus-host disease. Stem Cells (2013) 31(3):592–601. doi: 10.1002/stem.1314 23307526

[156] Moll G Ignatowicz L Catar R Luecht C Sadeghi B Hamad O. Different procoagulant activity of therapeutic mesenchymal stromal cells derived from bone marrow and placental decidua. Stem Cells Dev (2015) 24(19):2269–79. doi: 10.1089/scd.2015.0120 26192403

[157] Erkers T Nava S Yosef J Ringdén O Kaipe H. Decidual stromal cells promote regulatory T cells and suppress alloreactivity in a cell contact-dependent manner. Stem Cells Dev (2013) 22(19):2596–605. doi: 10.1089/scd.2013.0079 23701127

[158] Karlsson H Erkers T Nava S Ruhm S Westgren M Ringdén O. Stromal cells from term fetal membrane are highly suppressive in allogeneic settings in vitro. Clin Exp Immunol (2012) 167(3):543–55. doi: 10.1111/j.1365-2249.2011.04540.x PMC3374287 22288598

[159] Chang CJ Yen ML Chen YC Chien CC Huang HI Bai CH. Placenta-derived multipotent cells exhibit immunosuppressive properties that are enhanced in the presence of interferon-gamma. Stem Cells (2006) 24(11):2466–77. doi: 10.1634/stemcells.2006-0071 17071860

[160] Roelen DL van der Mast BJ in't Anker PS Kleijburg C Eikmans M van Beelen E. Differential immunomodulatory effects of fetal versus maternal multipotent stromal cells. Hum Immunol (2009) 70(1):16–23. doi: 10.1016/j.humimm.2008.10.016 19010366

[161] Croxatto D Vacca P Canegallo F Conte R Venturini PL Moretta L. Stromal cells from human decidua exert a strong inhibitory effect on NK cell function and dendritic cell differentiation. PloS One (2014) 9(2):e89006. doi: 10.1371/journal.pone.0089006 24586479 PMC3930605

[162] Sadeghi B Moretti G Arnberg F Samén E Kohein B Catar R. Preclinical toxicity evaluation of clinical grade placenta-derived decidua stromal cells. Front Immunol (2019) 10(2685). doi: 10.3389/fimmu.2019.02685 PMC6877599 31803191

[163] Sadeghi B Witkamp M Schefberger D Arbman A Ringden O. Immunomodulation by placenta-derived decidua stromal cells. Role of histocompatibility, accessory cells and freeze-thawing. Cytotherapy (2022) 25(1):68–75. doi: 10.1016/j.jcyt.2022.10.004 36333233

[164] Erkers T Solders M Verleng L Bergström C Stikvoort A Rane L. Frontline Science: Placenta-derived decidual stromal cells alter IL-2R expression and signaling in alloantigen-activated T cells. J Leukocyte Biol (2017) 101(3):623–32. doi: 10.1189/jlb.5HI0616-284R 27651429

[165] Fischer UM Harting MT Jimenez F Monzon-Posadas WO Xue H Savitz SI. Pulmonary passage is a major obstacle for intravenous stem cell delivery: the pulmonary first-pass effect. Stem Cells Dev (2009) 18(5):683–92. doi: 10.1089/scd.2008.0253 PMC3190292 19099374

[166] Sadeghi B Remberger M Gustafsson B Winiarski J Moretti G Khoein B. Long-term follow-up of a pilot study using placenta-derived decidua stromal cells for severe acute graft-versus-host disease. Biol Blood Marrow Transplant (2019) 25(10):1965–9. doi: 10.1016/j.bbmt.2019.05.034 31173898

[167] Ringden O Baygan A Remberger M Gustafsson B Winiarski J Khoein B. Placenta-derived decidua stromal cells for treatment of severe acute graft-versus-host disease. Stem Cells Transl Med (2018) 7(4):325–31. doi: 10.1002/sctm.17-0167 PMC5866941 29533533

[168] Baygan A Aronsson-Kurttila W Moretti G Tibert B Dahllof G Klingspor L. Safety and side effects of using placenta-derived decidual stromal cells for graft-versus-host disease and hemorrhagic cystitis. Front Immunol (2017) 8:795. doi: 10.3389/fimmu.2017.00795 28744284 PMC5504152

[169] Ringdén O Gustafsson B Sadeghi B. Mesenchymal stromal cells in pediatric hematopoietic cell transplantation a review and a pilot study in children treated with decidua stromal cells for acute graft-versus-host disease. Front Immunol (2020) 11:567210. doi: 10.3389/fimmu.2020.567210 33193339 PMC7604265

[170] Erkers T Kaipe H Nava S Mollden P Gustafsson B Axelsson R. Treatment of severe chronic graft-versus-host disease with decidual stromal cells and tracing with (111)indium radiolabeling. Stem Cells Dev (2015) 24(2):253–63. doi: 10.1089/scd.2014.0265 PMC4291217 25162829

[171] Ringdén O Solders M Erkers T Nava S Molldén P Hultcrantz M. Successful reversal of acute lung injury using placenta-derived decidual stromal cells. J Stem Cell Res Ther (2014) 4(10.4172):2157–7633.1000244. doi: 10.4172/2157-7633.1000244

[172] Sadeghi B Roshandel E Pirsalehi A Kazemi S Sankanian G Majidi M. Conquering the cytokine storm in COVID-19-induced ARDS using placenta-derived decidua stromal cells. J Cell Mol Med (2021) 25(22):10554–64. doi: 10.1111/jcmm.16986 PMC8581334 34632708

[173] Sadeghi B Ersmark B Moretti G Mattsson J Ringdén O. Treatment of radiculomyelopathy in two patients with placenta-derived decidua stromal cells. Int J Hematol (2020) 111(4):591–4. doi: 10.1007/s12185-019-02804-w PMC7102257 31853810

[174] Arnberg F Lundberg J Olsson A Samén E Jaff N Jussing E. Intra-arterial administration of placenta-derived decidual stromal cells to the superior mesenteric artery in the rabbit: distribution of cells, feasibility, and safety. Cell transplantation. (2016) 25(2):401–10. doi: 10.3727/096368915X688191 25976072

[175] Schrepfer S Deuse T Reichenspurner H Fischbein MP Robbins RC Pelletier MP. Stem cell transplantation: the lung barrier. Transplant Proc (2007) 39(2):573–6. doi: 10.1016/j.transproceed.2006.12.019 17362785

[176] Sadeghi B Ringden O Gustafsson B Castegren M. Mesenchymal stromal cells as treatment for acute respiratory distress syndrome. Case Reports following hematopoietic cell transplantation and a review. Front Immunol (2022) 13:963445. doi: 10.3389/fimmu.2022.963445 36426365 PMC9680556

[177] Ringden O Moll G Gustafsson B Sadeghi B. Mesenchymal stromal cells for enhancing hematopoietic engraftment and treatment of graft-versus-host disease, hemorrhages and acute respiratory distress syndrome. Front Immunol (2022) 13:839844. doi: 10.3389/fimmu.2022.839844 35371003 PMC8973075

[178] Ringden O Sadeghi B Moretti G Finnbogadottir S Eriksson B Mattsson J. Long-term outcome in patients treated at home during the pancytopenic phase after allogeneic haematopoietic stem cell transplantation. Int J Hematol (2018) 107(4):478–85. doi: 10.1007/s12185-017-2363-5 29143281

